# 3D modelling and simulation of the impact of wearing a mask on the dispersion of particles carrying the SARS-CoV-2 virus in a railway transport coach

**DOI:** 10.1038/s41598-023-35025-7

**Published:** 2023-06-01

**Authors:** Patrick Armand, Jérémie Tâche

**Affiliations:** 1 CEA, DAM, DIF, F-91297 Arpajon, France; 2 FLUIDIAN, F-95450 Commeny, France

**Keywords:** Computational science, Software, Fluid dynamics

## Abstract

Even though the Covid-19 pandemic seems to be stagnating or decreasing across the world, a resurgence of the disease or the occurrence of other epidemics caused by the aerial dissemination of pathogenic biological agents cannot be ruled out. These agents, in particular the virions of the Covid-19 disease, are found in the particles originating from the sputum of infected symptomatic or asymptomatic people. In previous research, we made use of a three-dimensional Computational Fluid Dynamics (CFD) model to simulate particle transport and dispersion in ventilated semi-confined spaces. By way of illustration, we considered a commuter train coach in which an infected passenger emitted droplets (1 and 10 µm) and drops (100 and 1000 µm) while breathing and coughing. Using an Eulerian approach and a Lagrangian approach, we modelled the dispersion of the particles in the turbulent flow generated by the ventilation of the coach. The simulations returned similar results from both approaches and clearly demonstrated the very distinct aerodynamics of the aerosol of airborne droplets and, at the other end of the spectrum, of drops falling or behaving like projectiles depending on their initial velocity. That numerical study considered passengers without protective masks. In this new phase of research, we first used literature data to develop a model of a typical surgical mask for use on a digital manikin representing a human. Next, we resumed the twin experiment of the railway coach, but this time, the passengers (including the infected one) were provided with surgical masks. We compared the spatial and temporal distributions of the particles depending on whether the spreader passenger wore a mask at all, and whether the mask was perfectly fitted (without leaks) or worn loosely (with leaks). Beyond demonstrating the obvious value of wearing a mask in limiting the dissemination of particles, our model and our simulations allow a quantification of the ratio of particles suspended in the coach depending on whether the infected passenger wears a mask or not. Moreover, the calculations carried out constitute only one illustrative application among many others, not only in public transport, but in any other public or private ventilated space on the basis of the same physical models and digital twins of the places considered. CFD therefore makes it possible to estimate the criticality of the occupation of places by people with or without a mask and to recommend measures in order to limit aerial contamination by any kind of airborne pathogen, such as the virions of Covid-19.

## Introduction

Since mid-2021, global concerns appear to be focusing on scourges other than the Covid-19 pandemic. This disease could nonetheless go through seasonal resurgences as do other respiratory infections, especially the flu. Covid-19 is transmitted mainly through contact with soiled, previously contaminated objects (also referred to as “fomites”) and through the transport and dispersion of particles emitted by infected people. In this regard, it has been established that virions, the extracellular form of viruses, are present and pathogenic in liquid particles produced by infected people when they sneeze or cough, but also when they speak or simply breathe. Virions are found in the sputum of symptomatic people, but also in that of asymptomatic people, who can unknowingly transmit the infection to others who, in turn, will be unaware that they have been contaminated. Airborne transmission of infectious agents carried in liquid particles of different sizes is discussed later in the introduction. It raises concerns, especially since it is not specific to Covid-19; indeed, it is common in many other respiratory diseases such as the other severe acute respiratory syndromes (SARS), Middle East respiratory syndrome (MERS) or the many types of influenza (H1N1) and their variants.

The sizes of particles expectorated depend on the dissemination event, and even on the human behind it (in other words, two people do not produce the same spectrum of liquid particles when breathing or coughing). The range of sizes extends over several orders of magnitude (from 0.1 to 1000 µm in aerodynamic diameter). A cut-off diameter (around 5 to 10 µm) separates the finer particles, which are “real” aerosols sustainably suspended in the air, from the larger particles, which, according to the conditions of their emission, either settle almost immediately on accessible surfaces or behave like projectiles. In this article, we consider particles of discrete sizes and use the word “droplets” for particles whose diameter is either 1 or 10 µm, whereas the word “drops” refers to particles with a diameter of either 100 or 1000 µm. Also, the word “diameter” implicitly means “aerodynamic diameter.”

On a global level, not all countries agree on the measures to be taken in the face of Covid-19: some apply strict testing and lock-down measures, while others put up with the presence of the disease as long as it remains limited. However, the World Health Organization (WHO) like the health institutions of many countries ended up advocating the mask use as valuable for limiting the dissemination of virions exhaled by infected people during respiratory events. Mask use was systematically made compulsory during the acute phases of the pandemic. It has persisted in places such as hospitals, pharmacies and public transport. Even though mask use currently remains compulsory only in certain countries, it will clearly become advisable again in the event of a Covid-19 resurgence, wherever it may occur.

In a previous article^1^, we attempted to fully demonstrate the value of Computational Fluid Dynamics (CFD) to account for the three-dimensional space and time dispersion of particles emitted by people infected with diseases leading to expectoration of pathogens such as virions. To illustrate our point, we studied the risk of Covid-19 transmission among public transport passengers. We created a twin experiment by reproducing the numerical mock-up of a commuter train car in which human manikins were placed. We assumed that an infected individual emitted droplets and drops while breathing and during coughing episodes. The particles were transported by the ventilation system of the coach and exhibited totally different aerodynamic behaviour depending on whether they were droplets (which perfectly followed the streamlines) or drops (which separated from the carrier fluid by their inertia and tended to sediment in the immediate vicinity of the spreader). In addition, the cough was characterised by the initial momentum given to the emitted particles. While the droplets adapted very quickly to the flow around them, this was not the case for the largest drops, which adopted a ballistic behaviour. This phenomenology was highlighted in our three-dimensional simulations, which examined the turbulent flow within the coach and the dispersion of particles of different sizes (using an Eulerian approach and a Lagrangian approach that led to similar results).

While our article^1^ enabled us to present and validate our CFD model, it was limited to the case in which passengers did not wear masks. This situation corresponds to what prevails today in places where the Covid-19 outbreak seems to be behind us, at least in some countries. When the epidemic was active, however, many governments mandated the wearing of masks on public transport; this remains the case in some parts of the world and could become widespread again in the event of a resurgence of Covid-19 or other respiratory diseases. In addition, we thought it would be interesting and useful to attempt to carry out CFD simulations featuring mask use by passengers. We were particularly interested in knowing if mask use could effectively reduce the dissemination of virions in a public space such as a railway coach. This led us to undertake simulations involving, once again, a twin experiment in a railway coach, this time with passengers wearing masks and, among them, one passenger assumed to be infected with the Covid-19 disease. In the simulations reported in this article as in the previous one, the liquid particles are assumed not to evaporate and we study their spatial and temporal distribution around the human manikins occupying the railway coach.

This new research article is structured as follows. We first present a review of the literature: on one hand, we take stock of what is known or still debated at the end of 2022 regarding the transmission of the SARS-CoV-2 virus; on the other hand, we examine the influence of mask use on the droplets and drops produced by an individual breathing and coughing. As there are many types of masks, special emphasis is placed on the surgical mask, which is very widespread due to its particularly low cost. We then devote a part of the article to the results of our modelling and simulation work. First we consider the head and bust portion of a human manikin, which is immersed in a motionless atmosphere; this allows us to examine the situations in which the manikin wears a tight-fitting mask, a loose-fitting mask, or no mask at all. We next present results obtained with complete human manikins wearing masks and placed in a commuter train, with one of the passengers being infected with the Covid-19 disease. In the next section of the article, we present a general discussion about the results obtained and the perspectives offered by our numerical approach in terms of scientific developments and operational applications. Part of the article is devoted to the methods used in the numerical study. In particular, we explain our choices regarding the production of droplets and drops, depending on whether the manikins wear more or less well-fitting masks, and regarding the aerodynamic conditions in the railway coach. We also present the CFD tool implemented in the study, as well as the computational resources and the associated computation times.

### Aerial transmission of the SARS-CoV-2 virus and other pathogenic respiratory agents

The mode of transmission of the SARS-CoV-2 virus (which causes Covid-19) was intensely debated in 2020, as the results were to determine the healthcare responses needing to be made. In July 2020, the WHO^2^ recognised that the SARS-CoV-2 virus could be transmitted from person to person through the air. This virus is known to be carried in liquid particles exhaled through the mouth and the nose, particularly when coughing, sneezing, speaking, singing or breathing^3^. These particles, whose exact chemical composition remains unclear, contain multiple virions of about 100 nm in size^4^. The combination of entrainment by the airflow, particle inertia, gravity and evaporation determines the evolution of the exhaled particles.

Historically, particles carrying virions have been separated into two categories according to their aerodynamic behaviour^5^, on the grounds that this dichotomy should be a source of guidance for national health authorities and the WHO. We therefore make a distinction between drops – “visible” particles with a diameter greater than about 5 to 10 µm, which fall under the effect of gravity without having time to evaporate, finally settling on exposed surfaces (fomites) – and droplets, presenting a diameter of less than 5 to 10 µm, which evaporate more or less rapidly to a dry nucleus and remain suspended in the air in the form of an aerosol^6^. The droplets are carried by the airflow, which depends on the local ventilation conditions^7^. They are likely both to cause contamination at longer distances and to penetrate deeper into the respiratory tract in comparison to drops^8,9^. The threshold of 5 to 10 µm that is usually considered has been discussed and questioned during the pandemic^3^**,** and it is clear today that all classes of particles must be taken into account, as well as the two modes of transmission at short and long distances^10^.

The number and size of particles exhaled by a spreader are highly variable. The overall exhalations of a human being are known to contain particles between 0.1 and 1000 µm in aerodynamic diameter, i.e. five orders of magnitude^11^. Symptomatic and asymptomatic carriers do not a priori produce the same number or the same size of viral particles. In addition, symptomatic carriers do not necessarily excrete higher viral-load drops and droplets than do asymptomatic infected people^12^. There are also people called “super-spreaders.” It has been shown, for example, that some individuals produce seventeen times more droplets during a cough compared to other individuals^13^. It has also been shown that the viral load of the particles changes according to the stage of the disease.

The proportion between exhaled drops and droplets is variable and still subject to debate, as is the potential for aerosol contamination. For example, trials^7^ have shown that 20,000 particles between 0.8 and 5.5 µm, along with 100,000 virions, are emitted every minute during speech. In a series of analyses, aerosols smaller than 5 µm have been shown to contain more SARS-CoV-2 virions than do particles larger than 5 µm^14^, while other findings tend to go the other way^13^. The number of exhaled particles varies depending on whether we consider a low-frequency event or a cyclic event. For example, a sneeze can produce around 10,000 particles^15^, a cough around 10 to 100 times fewer^16^ and breathing or speaking a minimum of 50 particles per second, but since breathing and speaking are recurrent phenomena, they are probably ten times more important in contamination than coughing or sneezing^17^.

Experimental work^13^ makes it possible to assess both the number of particles produced during coughing and speaking and the corresponding viral load. For instance, this work mentions that during a cough, 98% of the volume of particles is made up of drops of 100 to 1000 µm, with more than 20 10^6^ droplets (with a diameter of less than 10 µm) being produced in a single cough. By comparison, the experiment proposed for speech (“stay healthy” pronounced 10 times) produces more than 7 10^6^ droplets. The authors use a viral load estimate of 7 10^6^ virion copies per millilitre of respiratory sample. Measurement of the volume of the cough droplets shows that it has about 10^4^ copies, i.e. one in 2000 droplets contains at least one virion.

Finally, it should be noted that the diameter of the particles varies in the air under the effect of evaporation. The final particle diameter depends on many factors such as initial size, relative humidity, temperature, ventilation flows and residence time^11^. For example, an average particle size of 2 to 3 µm can be obtained for an initial size of 10 µm^18^.

While numerous scientific works carried out during the Covid-19 pandemic have supplemented the knowledge acquired over a long period of time on the transmission of infectious agents, many questions about the SARS-CoV-2 virus are not yet clearly resolved, such as the relative contagiousness of drops and droplets according to their diameters, the “minimum dose” to risk contamination, the number of virions exhaled by infected people or the evolution of the pathogenicity of the virions embedded in evaporating drops and droplets.

### Use of surgical masks and their effect on aerial transmission

The surgical mask is a single-use respiratory mask whose purpose is to limit to the immediate environment the spread of bacteria and viruses exhaled from the respiratory tract (mouth and nose) of the wearer. Its main purpose is to filter the largest respiratory drops (above a few tens of micrometers). Originally, this type of mask was worn by healthcare professionals during surgery to protect the sterile operating field and the patient receiving care. It is also worn by patients with a disease whose contagious agent is airborne.

When used correctly, a surgical mask quite successfully contains the dispersion of respiratory drops produced during a sneeze or a cough. It is commonly accepted to be an effective device for blocking drops projected by the wearer and measuring several tens of microns. It has been shown to greatly limit the transmission of airborne viruses (influenza, coronavirus, etc.) by infected people^19^. That said, it is not very effective in stopping the transmission of fine aerosols smaller than 5 µm^20^, and its effectiveness depends on its design, the materials used in its manufacture, its dimensions and its fit on the face.

Above all, the surgical mask protects the individuals surrounding the person wearing it – but the wearer is also protected from projections of drops, though it is unknown to what extent exactly. The protection provided by a surgical mask during inhalation is real, but unquantified and extremely variable. Such a mask is not designed to protect the wearer from inhaling airborne bacteria or viral particles.

Generally speaking, filtering facepiece (FFP) masks are personal respiratory equipment defined by standards such as EN 149^21^ in the European Union. This type of mask protects the wearer of the mask against the inhalation of particles in suspension in the air (average aerosol diameter of 0.6 μm) and drops of larger diameters. Leaks inside the mask are also standardised. There are several types of masks – FFP1, FFP2 and FFP3 – categorized by their filtration of aerosols with an average diameter of 0.6 µm (respectively 80%, 94% and 99%) and their degree of leakage towards the inside of the masks (respectively less than 22%, 8% and 2%).

The surgical mask is not a filtering respiratory device and cannot be certified as such. To be approved, however, it must meet standardised criteria based on bacterial filtration efficiency (BFE; during exhalation only) and splash resistance. For instance, in the EU, types I and II correspond to masks with, respectively, BEF > 95% and BEF > 98% of an expired aerosol with an average diameter of 3 µm, while type III is like type II but is also resistant to splash. There exists a test protocol for evaluating BFE^22^ whose presentation would be beyond the scope of this article, as would be the description of all standards that apply to masks intended for workers (for instance, medical staff) or the general public. The reader is referred, for example, to an Internet site^23^ that provides an interesting compilation of the standards that apply in the USA, the EU, China, Japan, South Korea and elsewhere.

### Particle filtration efficiency during exhalation

Several authors have studied the filtration efficiency of surgical masks for various particle sizes, including fine particles. In one experiment^24^, different types of masks were tested with the assumption that they were perfectly fitted, i.e. without leakage between the mask and the wearer’s face. Drops with a diameter greater than 10 µm were generally filtered by the different masks, as were particles with a diameter less than 200 nm, due to the Brownian effect. Still, none of the masks tested, apart from FFP2 N95, could filter 100% of the droplets of intermediate size whose diameter was between 1 and 5 μm. In this experiment, even with a perfectly tight fit, aerosol leakage through the surgical mask represented 0.1% to 0.2% of the exhaled particles. Another experimental work^25^ involving different types of perfectly fitting surgical masks gave even poorer overall aerosol filtration efficiency results for surgical masks, with about 50% of particles with a diameter between 1 and 8 µm being retained.

In practice, leakage can be very significant at the wearer’s face, because the mask lets a large quantity of air pass around its perimeter. For instance, the presence of fog on eyeglasses shows that a good deal of air is exiting directly without passing through the filter screen. The problem of leaky surgical masks is not new, and it has often been studied already, at least qualitatively, leading to wear and adjustment recommendations for healthcare personnel^26^. A few precautions can limit the rate of leakage: these include a knot in the ear loops, a well-adjusted nose clip or the use of a cloth mask over the surgical mask, as per CDC recommendations^27^.

More recently, the problem of leakage from surgical masks has been presented experimentally in a relatively large number of scientific publications. For instance, an experimental reconstitution^28^ of the cough of a human manikin showed that only 56% of aerosols between 0.1 and 7 µm were filtered by a surgical mask, due to leaks around the edge of the mask. The filtration percentage increased to 77% with adjustment by side knots and the use of a nose clip, and even to 85% when the surgical mask was covered with a cotton mask.

Detailed CFD simulation work carried out on the subject in 2021 by the Riken Scientific Research Institute (Japan)^29^ also showed that the fraction of aerosols passing through different types of fabric masks without being filtered was larger than 70% with a variable fraction of aerosols leaking through the spaces between the wearer’s face and the mask.

Apart from its more or less effective filtration properties, a surgical mask can significantly reduce the airflow velocity during a sneeze, during a cough or within the respiratory cycle^30–34^. That said, the inhalation and exhalation phases also increase leaks on the perimeter of the mask due to “pumping” effects. Thus, the air jets resulting from these leaks can be highly turbulent and directional, which increases the effects of aerosol dispersion in the transverse directions but redirects the aerosols in directions that are a priori less problematic than a breath of air emitted directly from the mouth or nose of the wearer^35,36^.

It is also possible to visualise changes in exhaled airflows through density differences (due to temperature differences between the lukewarm exhalation and the ambient air) by means of the Schlieren process^37^. In the work cited, the authors showed that the direction and range of the exhaled airflow were modified according to the type of mask worn. Instead of passing through the filtering part of the mask, the air flows partially around the filtering part through leaks. Leakage can be two thirds of the total airflow through all parts of the mask. This fraction is much larger for surgical masks than, for example, FFP masks. The leaks between the mask and the face may be so significant that, according to the authors, the effectiveness of the masks should be considered based on the existence of secondary airflows around the perimeter of the mask, which depends on whether or not the mask is properly worn rather than on its intrinsic filtration efficiency or its ability to reduce the main airflow through the mask.

Looking beyond experimental evaluations, CFD presents the advantage of allowing precise access to airflow velocities and particle trajectories. That said, few simulations involving surgical masks and their inherent leaks have been carried out. One example is given by a CFD study^38^ of a human manikin wearing a surgical mask, in which the air and droplet leakage through and around the mask were evaluated for a five-second cyclical cough (with 1,008 droplets per cycle and a maximum expectoration velocity of 5 m.s^−1^). The numerical simulations were carried out with the Open FOAM software using an unsteady RANS approach for turbulence and a Lagrangian approach for particle tracking. The particle diameters were between 1 and 300 µm, and the filtration efficiency of the modelled surgical mask was assumed to be 91%. The authors used these simulations to identify the main locations of the leaks, evaluating the airflow velocities through these leaks to be approximately 0.2–0.4 m.s^−1^. The results also made it possible to estimate the relative proportions of droplets that were blocked by the mask, that passed through the mask and that escaped through leaks. The positive role of the mask, both in terms of filtration and reduced exhaled flow, was highlighted. Unfortunately, the authors did not establish a connection between the nature of the leakage and the particle diameter.

### Particle filtration efficiency during inhalation

There exist no standardised data on wearer protection against incoming aerosols (that can also penetrate inside the mask by passing through the spaces between the wearer’s face and the mask). There have, however, been experimental studies published on this subject^39–41^. Between 20 and 80% of aerosols with a diameter of less than 1 µm passed through the mask, depending on its design, the number of layers, the material used for filtration and the airflow imposed through the mask. In another experimental work^42^, 20% to 80% of 1– to 3–µm diameter droplets passed through the mask.

On the same topic, an interesting experimental comparison^43^ was made for different types of masks regarding their filtration efficiency during inhalation (inward) and exhalation (outward). The aerosol diameters were between 0.04 and 1 µm in the first case, and between 2 and 5 µm in the second. The filtration efficiency of the mask was evaluated by tests on a specific test bench using a sample of the mask material (which therefore presents no leaks). In addition, the inward and outward protection efficiencies were determined using tests on manikins (accounting for leaks). For diameters less than 5 µm, the results depend on the diameters, with the exhalation and inhalation filtration efficiencies found to be between 25 and 75%. There is a significant deficiency in the effectiveness of the surgical mask for diameters below 2 µm, whether the wearer is inhaling or exhaling. Above 5 µm, the exhalation and inhalation efficiencies of the surgical mask were comparable (around 75%).

Very few CFD simulations have focused on the filtration of inhaled aerosols through a surgical mask. The study^44^ considers the head of a human manikin inhaling aerosols through a perfectly fitted surgical mask (with no leaks). The aerosols were between 1 and 20 µm in diameter. The filtration efficiency of the mask was set to 65% for all diameters. The originality of this study resides in its consideration of both the upper airways (nose and pharynx) and the lower airways (mouth and larynx), with the results showing that mask use clearly alters the flow near the nose and mouth. The air velocities were significantly lower and the particles entered less deeply into the respiratory tract favouring the deposition of aerosols in the upper airways (nose). Overall, wearing the mask reduced the quantity inhaled by three and five, respectively, for the 3–10 µm and 15 µm aerosols. For the aerosols of 1–3 µm in diameter, the quantity inhaled was almost the same regardless of whether a mask was worn or not.

Another publication^35^ presents a CFD and experimental study featuring the head of a human manikin equipped with an FFP2 mask. Even though the mask studied was not a surgical mask, this study enabled the assessment of leak sites around the perimeter of the mask and the way in which these leaks were distributed. The results indicated that, on average, leaks occurred mainly at the level of the nose (35 to 50%), to a lesser extent near the cheeks (20 to 25%) and least of all near the chin (6 to 12%).

### Summary of the literature review focusing on surgical masks

A number of studies have examined the effectiveness of surgical masks, though most of them have not been carried out in the context of the Covid-19 pandemic, but instead for other infections (especially influenza). In addition, most of these studies have been experimental and solely qualitative, with different areas of focus (samples of filtering materials, masks placed on human manikins or patient cohorts). Due to differences among the protocols and the challenges involved in making the various measurements, the conclusions of these studies can be contradictory. Nevertheless, the following information can be derived about surgical masks:

- Filtration efficiency in experiments implying real people or human manikins is lower than that measured using devices for testing masks.
- A surgical mask contains the dispersion of respiratory drops of more than 10 µm in diameter according to standards. That said, it is much less effective in stopping the transmission of aerosols of less than 3 µm in diameter.
- For a perfectly fitted surgical mask, the overall filtration efficiency during exhalation is around 50% for 1–8 µm droplets^25^ or between 50 and 75% for droplets smaller than 2 µm^43^. Still, for 1–5 µm droplets, leakage is limited to 0.1 to 0.2%^24^.
- A surgical mask primarily protects those around the person wearing it. The wearer is also protected from projections of drops without it being known in which proportions exactly. The protection provided to the wearer during inhalation is not standardised. Experimental studies show that 20–100% of 1–3 µm particles pass through the mask^39–42,44^.
- In practice, leakage is significant, and the mask allows large quantities of air and large numbers of particles to pass around it. While these leaks are not precisely quantified, their location is relatively well known. We can retain the following results:
- During a cough, 56% of 0.1–7 µm droplets are blocked, while the others escape via side leaks^28^;
- During a cough, the fractions of droplets blocked by the mask, passing through it and escaping through the leaks have been evaluated, and the velocity through the leaks is 0.2–0.4 m.s^−1^^38^;
- In respiratory events, leaks around the edge of the mask are distributed on average as follows: 35 to 50% around the nose, 20 to 25% on each side of the cheeks, and 6 to 12% along the chin^35^;
- In respiratory events, the outward and inward filtration efficiencies of a surgical mask are between 25 and 75% for droplets of less than 5 µm in diameter, and they are of the same order for particles larger than 5 µm^43^.
- Apart from its filtration properties, a surgical mask can greatly reduce the velocity of the breath of air emitted during the respiratory cycle^30–32^.

## Results #1—Implementation of a manikin wearing a model of a mask

**Figure 1:**
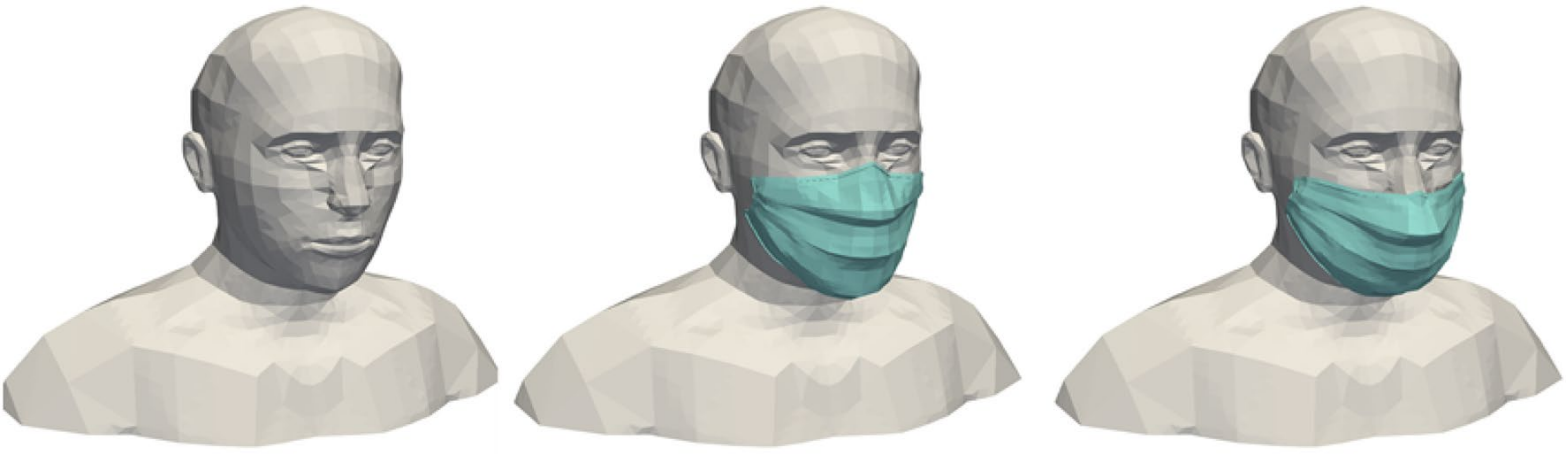
3D geometries of the human manikins in three different configurations: wearing no mask (left), wearing a mask perfectly fitted on the face (centre) and wearing a mask loosely with the presence of leaks (right). Images created by the authors with Paraview 5.8.1 (www.paraview.org).

This first series of simulation results concerns the implementation of the model developed to represent a human manikin (limited to a head and bust), either equipped with a surgical mask or not. Figure 1 shows the cases created for 3D modelling: in case #1, the manikin wears no mask; in case #2, the manikin wears a perfectly fitted mask (there are no leaks between the face and the mask); and in case #3, the manikin wears a mask loosely (there are leaks between the face and the mask). We note that the manikin’s facial features can be clearly seen; this model is therefore much more refined than the one used in our previous study^1^.

The effect of the mask on the processes of breathing or coughing is a key aspect of our numerical study. The presence of the mask or, more precisely, of its filtering part made of a porous media results in slowing and diffusing the emitted flow. This effect has been modelled by considering that the exhaled (or inhaled) air passes through the whole surface of the filtering part of the mask, which is much larger (by a factor of about 30) than the surface of the mouth (when no mask is worn). We have thus applied dedicated boundary conditions and not explicitly accounted for the porous medium constituting the filtering part of the mask.

**Figure 2:**
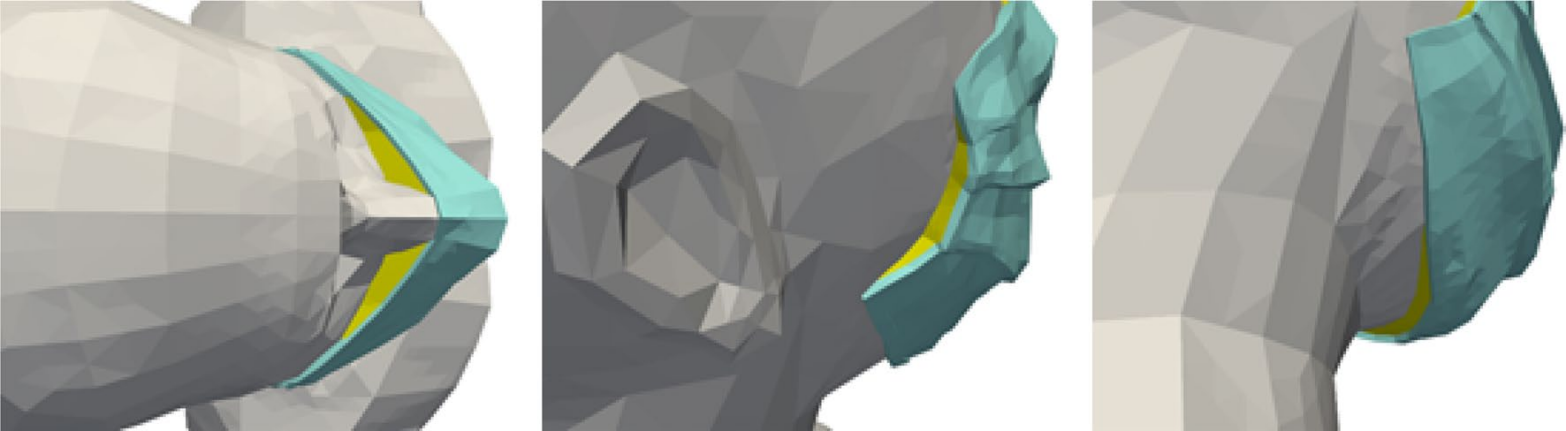
Location of the leak zones (in yellow) for the configuration with the mask incorrectly worn with leaks at the nose (left), cheeks (centre) and chin (right). Images created by the authors with Paraview 5.8.1 (www.paraview.org).

In case #1, the air inspired and expired by the manikin passes through its mouth, where the boundary condition of the simulation domain is located. In case #2 and case #3, we do not explicitly model the mask as such, nor consider what happens between the part of the manikin’s face covered by the mask and the mask itself (in other words, this part of space is not meshed). The boundary condition is located at the level of the filtering part of the mask, through which the exhaled air and the particles not retained by the mask enter the simulation domain (and the inhaled air and a few particles leave it). In case #2, in which the mask is perfectly adjusted, all the air and the particles not retained by the mask go through the filtering part of the mask. In case #3, in which the mask is worn loosely, the air and particles exit both from the filtering part of the mask and through localized leakage surfaces on the edges of the mask. Therefore, the boundary conditions are located on the one hand at the level of the filtering part of the mask, and on the other hand at the level of the leaks. Leaks are taken into account close to the nose, cheeks and chin, as shown in Fig. 2. The surface area of these leak zones has been established based on data in the literature^38^.

**Figure 3:**
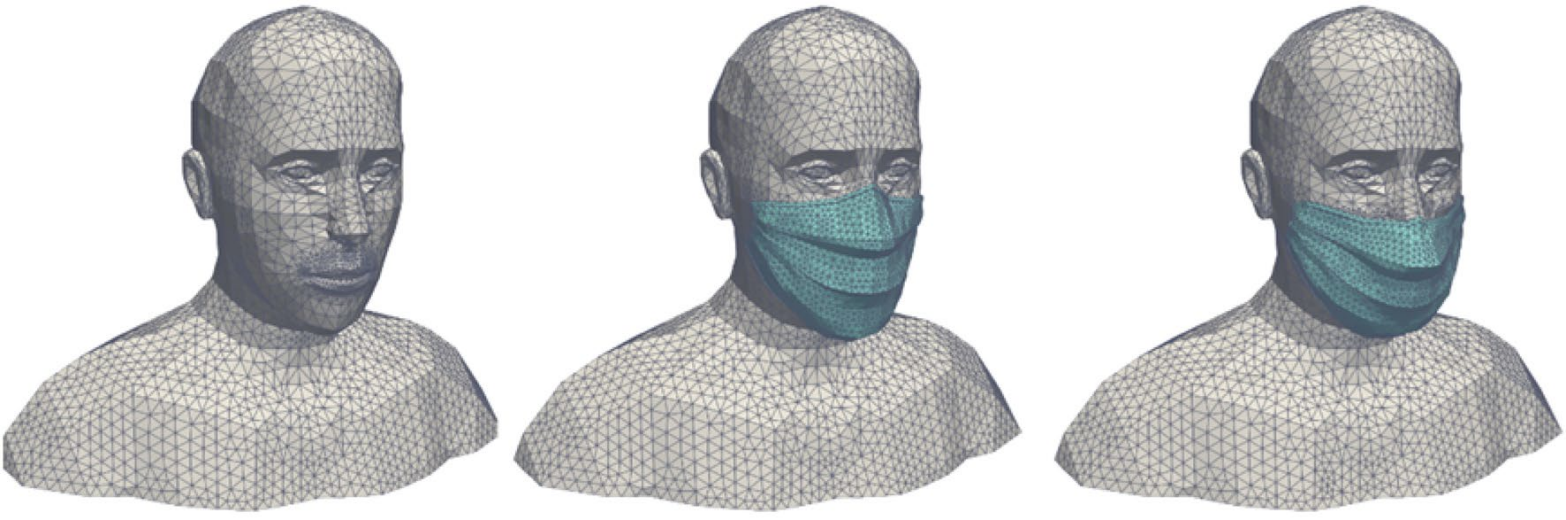
Surface meshes of the human manikins for the three different configurations: with no mask (left), with a perfectly worn mask (centre) and with an incorrectly worn, leaky mask (right). Images created by the authors with Paraview 5.8.1 (www.paraview.org).

The simulation domain around the head and bust of the manikin is a box whose boundaries are assumed to be at atmospheric pressure. The size of this box is chosen to be large enough to respect good CFD practices: 1.7 m long, 1.4 m wide and 0.7 m high. Figure 3 shows the meshes associated with the different ways of wearing the mask. These meshes consist of tetrahedral cells (about 500,000 cells in case #1 and 240,000 cells in case #2 and case #3), with the size of the cells on the surface of the manikin varying between 3 mm and 1 cm. The maximum cell size at the boundaries of the box is 5 cm. The skewness determines how close to ideal the mesh cells are (here, the ideal is to have equilateral tetrahedral cells). A value of 0 indicates an equilateral cell and a value of 1 a completely degenerate cell. Regarding the meshes around the bust and head of the human manikin (with or without a mask), at least 99.9% of the cells have a skewness of less than 0.85 and most of them have a minimal skewness of less than 0.001, which is a proof of the quality of the three meshes.

Coughing and breathing are typical events causing the dissemination of droplets and drops that are likely to carry the SARS-Cov-2 virus, as well as any other pathogenic biological agents. The dispersion of human sputum into the air is anticipated to be significantly different depending on whether the person wears a mask more or less properly or does not wear one at all, as will be shown in the following sections. For the results presented below, there is no flow driver other than the dissemination event (breathing or coughing). More details on the flow and dispersion calculations are given in the “Methods” section of this article.

### Dissemination of droplets generated by breathing

Hereafter, we present the results of the simulations with a breathing manikin. Each case of mask use is illustrated by the velocity fields as well as the distribution of the droplets (1 µm) in the computational domain at different times. The balance of droplets exhaled and inhaled by the manikin is also indicated.

#### Case #1—No mask

**Figure 4:**
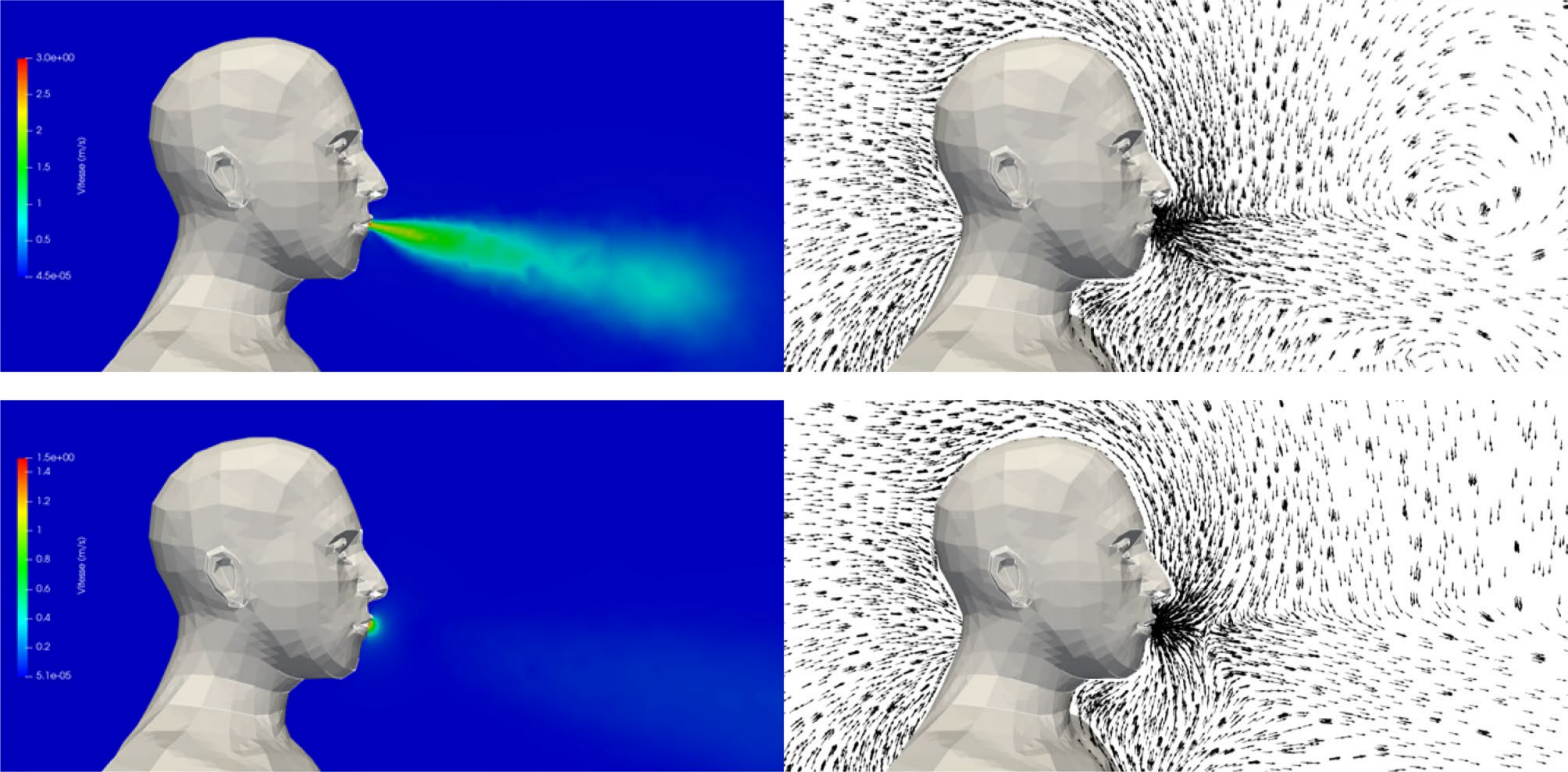
Case #1—No mask—Velocity modulus (left column) and velocity vectors (right column) during the exhalation phase (top row) and the inhalation phase (bottom row). Images created by the authors with Paraview 5.8.1 (www.paraview.org).

Figure 4 shows the velocity modulus and the velocity vectors in the plane of symmetry of the manikin during exhalation and during inhalation. We observe exhaled airflow at relatively high velocity in the first phase and air suction in the second phase. These phases are repeated every 5 s during the 120 s sequence.

**Figure 5:**
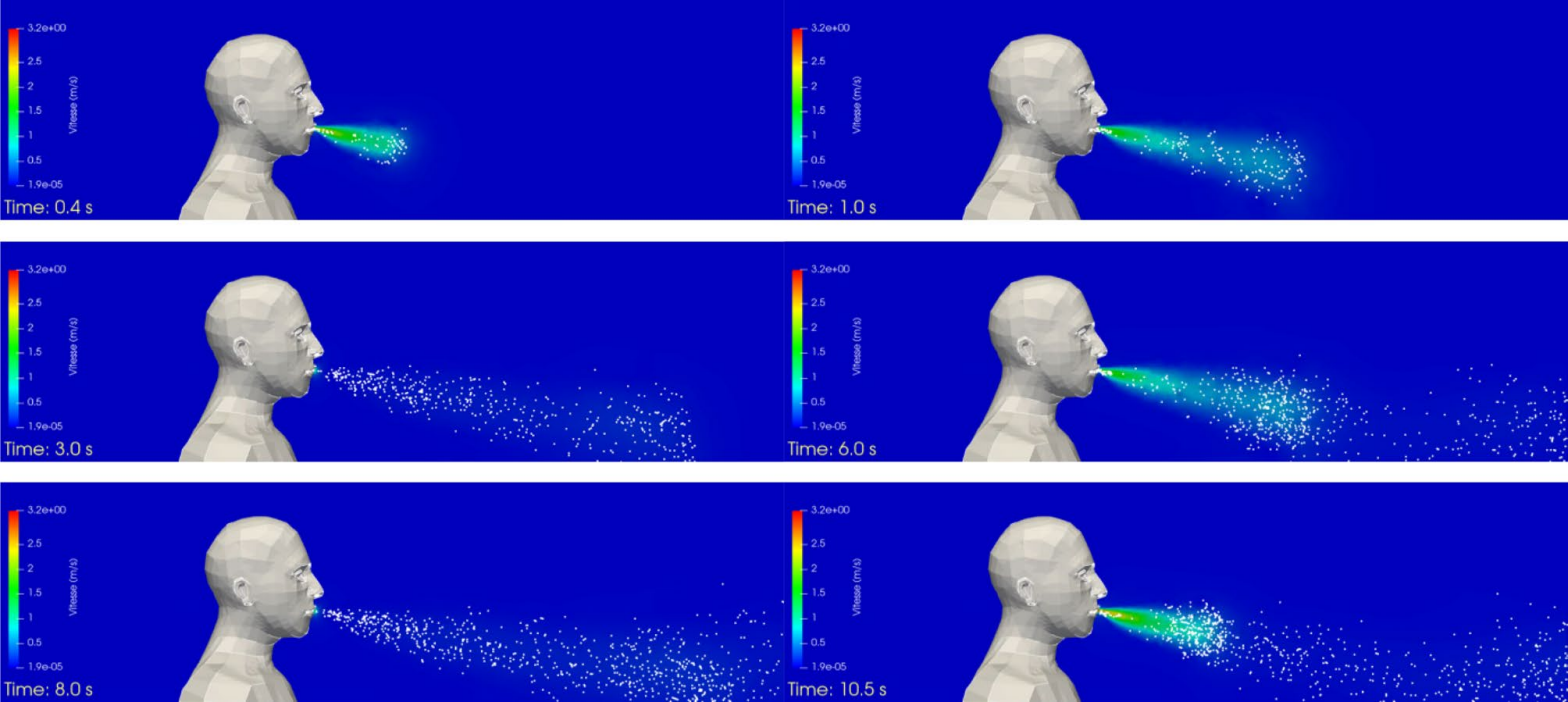
Case #1—No mask—Velocity field and distribution of the droplets in the plane of symmetry of the manikin at different times (left to right, and top to bottom): t = 0.4 s; 1 s; 3 s; 6 s; 8 s; and 10.5 s. Images created by the authors with Paraview 5.8.1 (www.paraview.org).

Figure 5 shows the velocity field and the droplets exhaled by the manikin (in its plane of symmetry) in the first instants of the calculation. The exhalation and inhalation phases are repeated successively throughout the 120 s sequence. Some droplets are sucked back into the manikin and are removed from the simulation, while droplets that reach one of the boundaries of the box exit the domain and are also removed from the simulation. The number of droplets that are exhaled by the manikin, i.e. injected into the simulation domain during the 120 s sequence, is equal to 25,113, and the number of droplets that are inhaled, i.e. re-aspirated through the manikin’s mouth during the 120 s sequence, is 2,756. This latter value represents approximately 11% of the total number of droplets.

#### Case #2—Perfectly fitted mask (no leaks)

**Figure 6:**
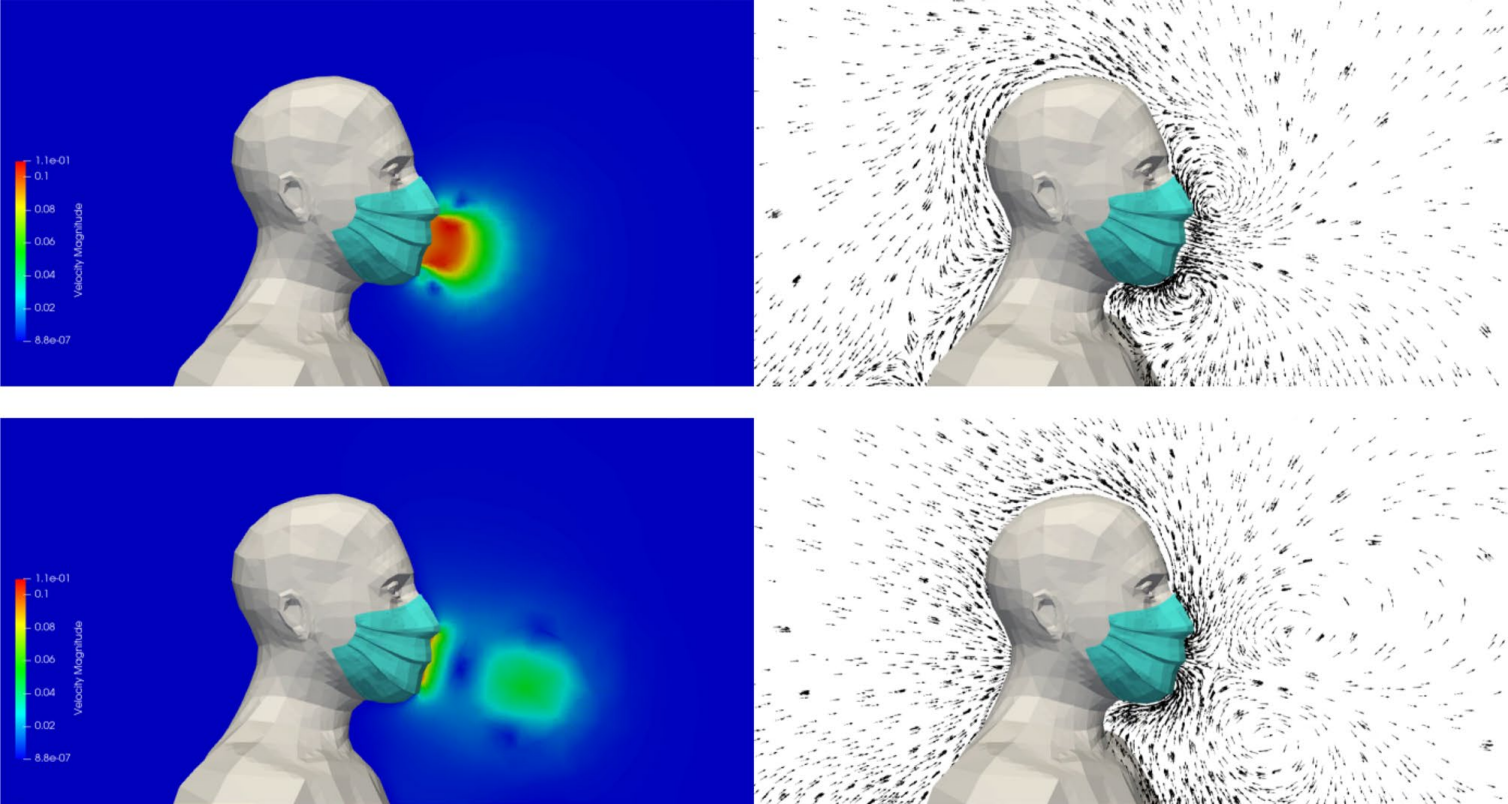
Case #2—Perfectly fitting mask—Velocity modulus (left column) and velocity vectors (right column) during the exhalation phase (top row) and the inhalation phase (bottom row). Images created by the authors with Paraview 5.8.1 (www.paraview.org).

Figure 6 shows the velocity modulus and the velocity vectors in the plane of symmetry of the manikin during exhalation and during inhalation. We note that the mask drastically reduces the velocity of the exhaled airflow. The maximum velocity upon expiration is of the order of 0.1 m.s^−1^ instead of 3 to 4 m.s^−1^ without a mask (see Fig. 4). The dynamics of the respiratory cycle being identical, the two phases of exhalation and inhalation are repeated, as for case #1, every 5 s during the simulated sequence of 120 s.

**Figure 7:**
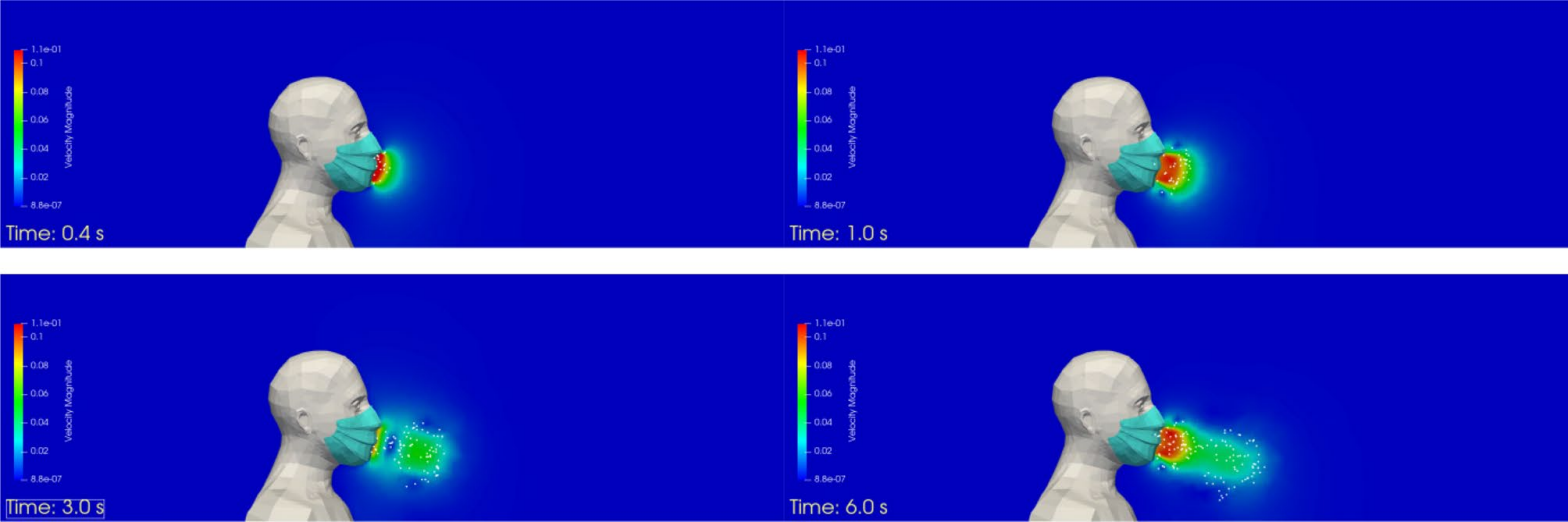
Case #2—Perfectly fitting mask—Velocity field and distribution of the droplets in the plane of symmetry of the manikin at different times (left to right, and top to bottom): t = 0.4 s; 1 s; 3 s; and 6 s. Images created by the authors with Paraview 5.8.1 (www.paraview.org).

Figure 7 shows the velocity field and the droplets exhaled by the manikin (in its plane of symmetry) at different times during the simulated respiratory cycle. As in case #1, some droplets are re-aspirated by the manikin and are removed from the simulation. The number of droplets here is, logically, much lower, because the surgical mask is hypothesised to exhibit a droplet filtration efficiency of 75%. The number of droplets that are exhaled by the manikin, i.e. injected into the simulation domain during the 120 s sequence, is equal to 6072, and the number of droplets that are inhaled, i.e. re-aspirated by the manikin during the 120 s sequence, is 1865. This latter value represents more than 30% of the total number of droplets, which is a much greater proportion than in the case where the manikin does not wear a mask. This is explained by the lower exhalation velocity through the mask, which moves the droplets a lesser distance away from the mask. These droplets are then more likely to be re-aspirated by the manikin during the next inhalation phase.

#### Case #3—Poorly fitted mask (with leaks)

**Figure 8:**
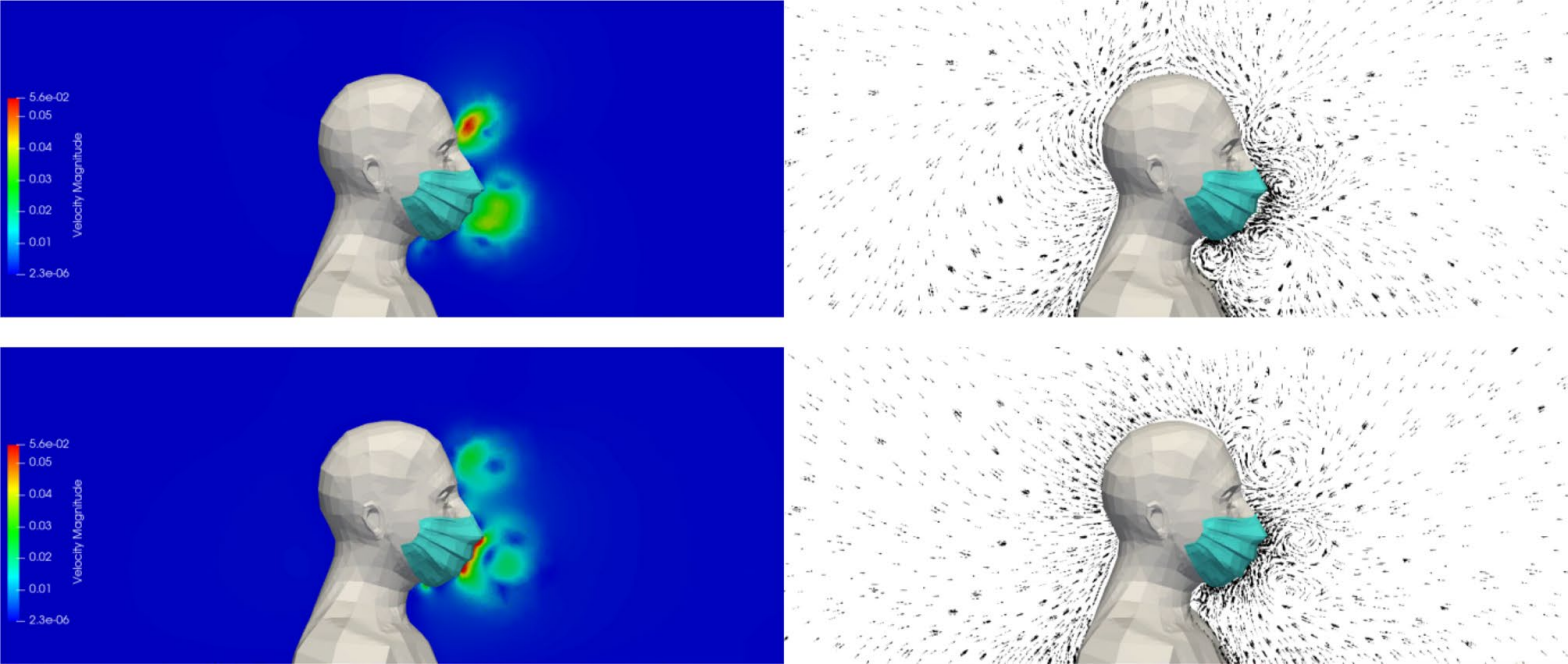
Case #3—Poorly fitting mask—Velocity modulus (left column) and velocity vectors (right column) during the exhalation phase (top row) and the inhalation phase (bottom row). Images created by the authors with Paraview 5.8.1 (www.paraview.org).

Figure 8 shows the velocity modulus and the velocity vectors in the plane of symmetry of the manikin during exhalation and during inhalation. In addition to the role of the mask already observed in case #2, involving the reduction of the velocity of the exhaled airflow, one can visualize the airflow coming from the leaky areas at the level of the nose, cheeks and chin. The dynamics of the respiratory cycle being identical to the previous cases, the two phases are repeated every 5 s during the simulated sequence of 120 s.

**Figure 9:**
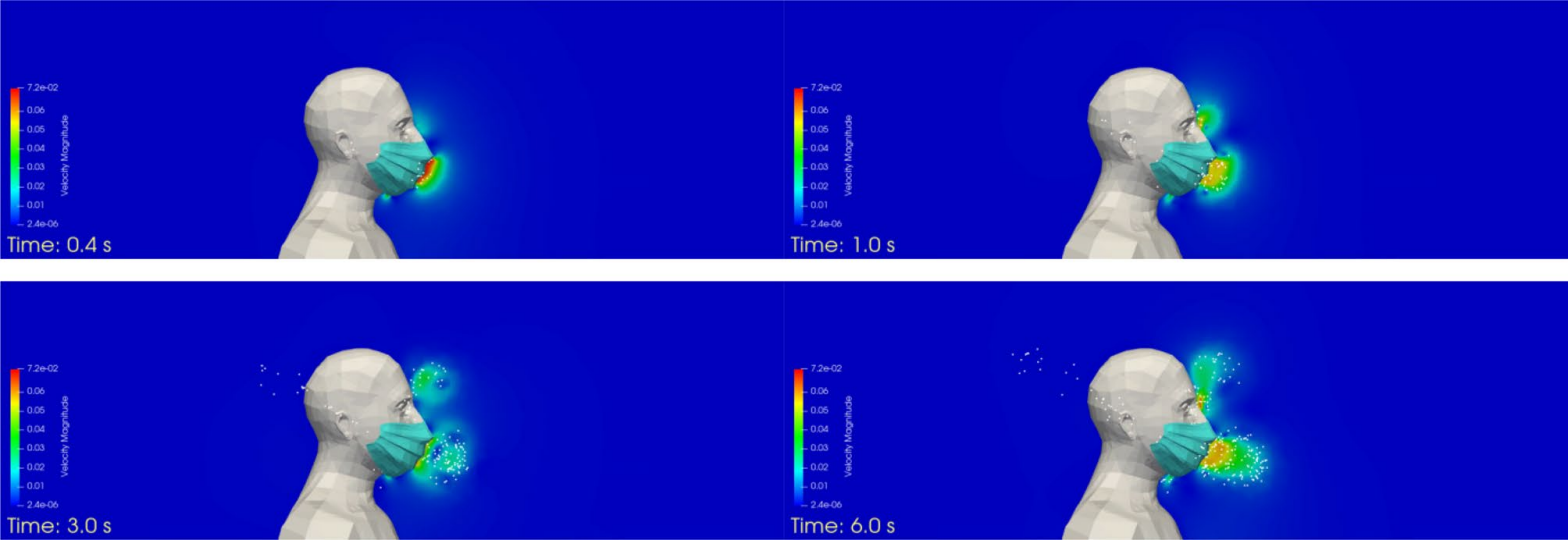
Case #3—Poorly fitting mask—Velocity field and distribution of the droplets in the plane of symmetry of the manikin at different times (left to right, and top to bottom): t = 0.4 s; 1 s; 3 s; and 6 s. Images created by the authors with Paraview 5.8.1 (www.paraview.org).

Figure 9 shows the velocity field and the droplets exhaled by the manikin (in its plane of symmetry) at different instants during the simulated respiratory cycle. The droplets come out through the mask and the leak zones. As in the previous cases, some droplets are sucked back by the manikin and are removed from the simulation. The number of droplets that are exhaled by the manikin, i.e. injected into the simulation domain during the 120 s sequence, is equal to 9963, and the number of droplets that are inhaled, i.e. re-aspirated through the mask and the leak zones (between the mask and the manikin’s face) during the 120 s sequence, is 2552. The total number of re-inhaled droplets represents more than 25% of the number of exhaled droplets, with most of this re-inhalation occurring through the mask (approximately 15%), and then through the right and left nose leaks (around 3% each), the chin leaks (around 2%), and, finally, the right and left cheek leaks (a bit more than 1%).

### Dissemination of droplets and drops by a cough

Hereafter, we present the results of the simulations with a coughing manikin. For the sake of brevity, we will describe only the cases in which the manikin either does not wear a mask or wears a mask loosely. Each of these two cases is illustrated by the distribution of the droplets (1 µm and 10 µm) and the drops (either 50 µm or 100 µm, and 1000 µm) in the computational domain around the manikin at different times.

#### Case #1—No mask

**Figure 10:**
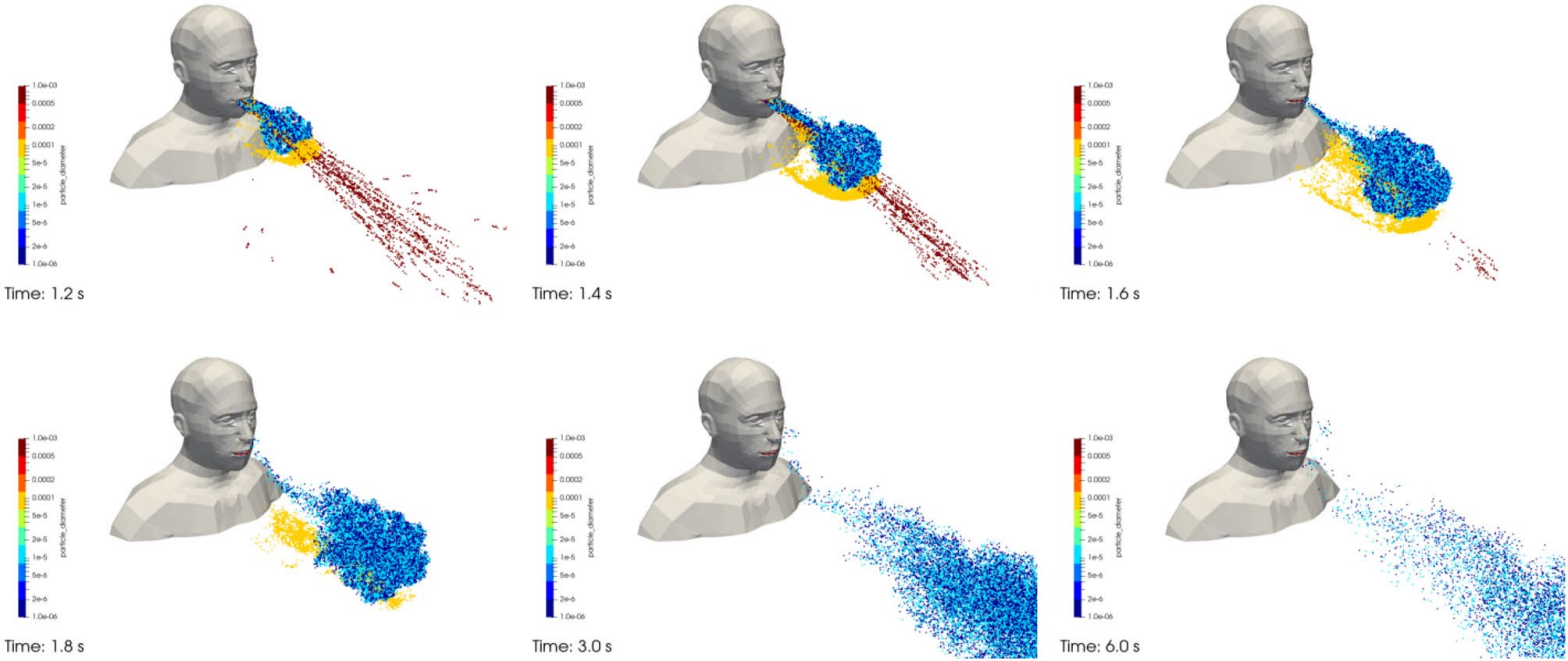
Case #1—No mask—Drops and droplets projected during coughing at different times (left to right, and bottom to top): (t = t_0_ + 0.2 s; t_0_ + 0.4 s; t_0_ + 0.6 s; t_0_ + 0.8 s; t_0_ + 2 s; and t_0_ + 5 s). Dark blue, light blue, yellow and red dots respectively represent particles of 1 µm, 10 µm, 100 µm and 1,000 µm in diameter. Images created by the authors with Paraview 5.8.1 (www.paraview.org).

Figure 10 shows the droplets of 1 µm (dark blue dots) and 10 µm in diameter (light blue dots) as well as the drops of 100 µm (yellow dots) and 1000 µm in diameter (red dots) that are exhaled by the manikin at different times during the simulation. The cough of the manikin starts at t_0_ = 1 s. We note the behaviour already observed during previous work for the drops of 100 µm or 1000 µm in diameter, namely deposition in the close vicinity of the manikin or ballistic trajectories that essentially result from inertial and gravitational effects. The droplets with a diameter of 1 µm or 10 µm behave identically overall; they are transported by the airflow and remain sustainably suspended in it, as is typical for an aerosol. When the particles reach one boundary of the box encompassing the manikin, they are removed from the simulation.

#### Case #3—Poorly fitted mask (with leaks)

**Figure 11:**
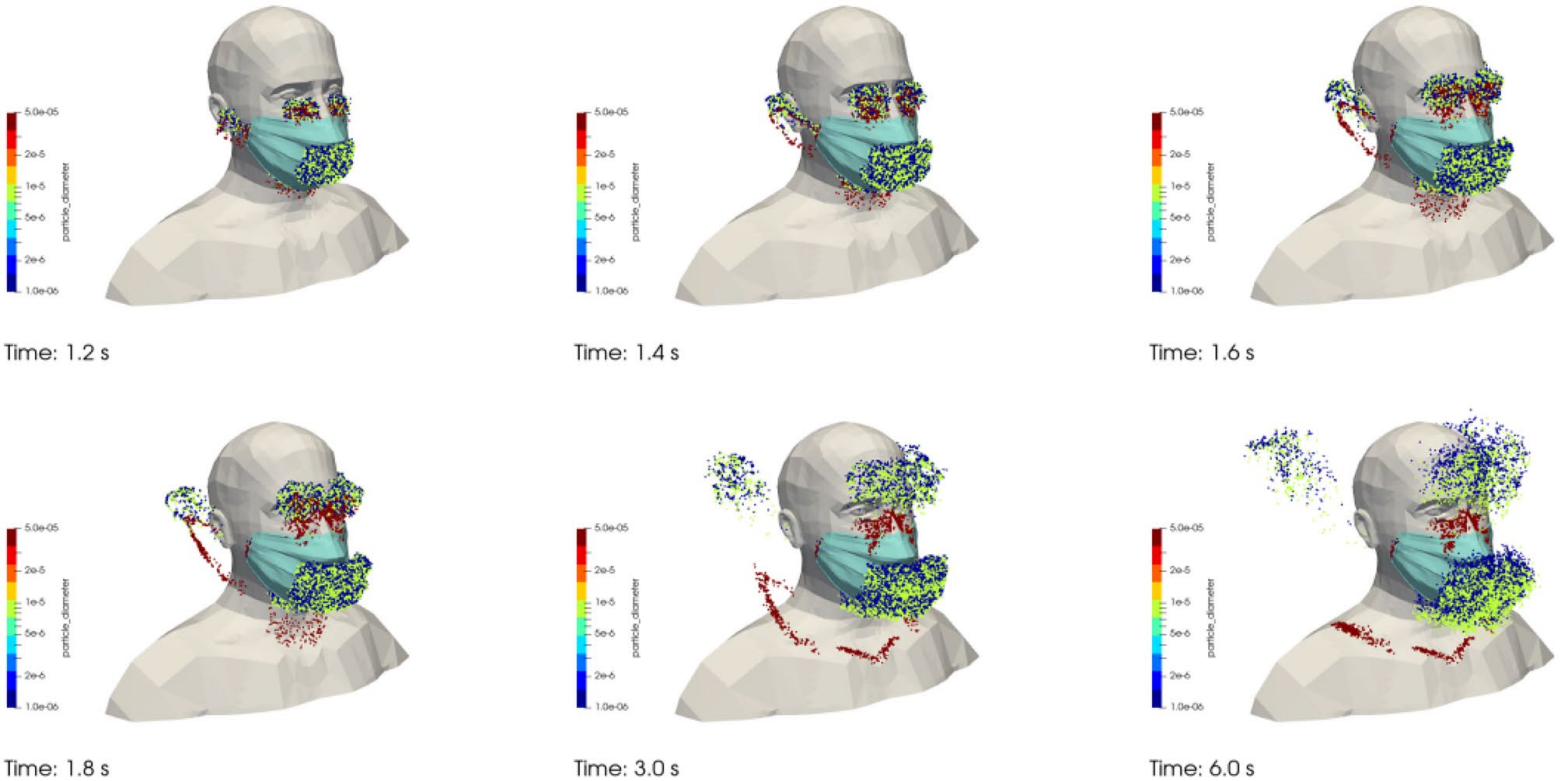
Case #3—Poorly fitting mask—Drops and droplets projected during coughing at different times (left to right, and bottom to top): (t = t_0_ + 0.2 s; t_0_ + 0.4 s; t_0_ + 0.6 s; t_0_ + 0.8 s; t_0_ + 2 s; and t_0_ + 5 s). Dark blue, green and red dots respectively represent particles of 1 µm, 10 µm and 50 µm in diameter. Images created by the authors with Paraview 5.8.1 (www.paraview.org).

Figure 11 shows the droplets of 1 µm (dark blue dots) and 10 µm in diameter (green dots) as well as the drops of 50 µm in diameter (red dots) that are exhaled by the manikin at different times during the simulation. The cough of the manikin starts at t_0_ = 1 s. While the 50 µm drops settle quickly on the face and shoulders of the manikin after being exhaled, the 1 µm and 10 µm droplets behave like aerosols. Because the latter are not projected far from their source, they mostly remain in the immediate vicinity of the face of the manikin in the absence of external flow.

## Results #2—Application to the digital twin of a railway coach and its passengers

Throughout the world, regional rail transport networks, as well as metro networks in urban areas, bring millions of passengers into daily contact with each other. There is therefore a strong interest in examining how an airborne pathogen present in the particles emitted by infected passengers (whether or not they wear a facial mask according to the rules in force locally) can spread in the semi-confined, ventilated space of a railway coach. This is why we chose this type of application to carry out CFD simulations representing the dispersion of Covid-19 virions during respiratory events involving infected people wearing or not wearing a mask.

**Figure 12:**
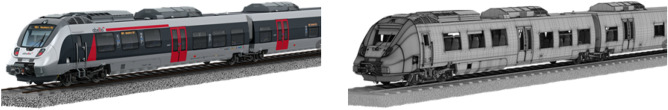
Model of the train chosen in the CFD study (left) and its triangulated surface (right).

There are few public data on the actual geometry of railway coaches. In order to create the digital twin of a typical commuter train and represent its geometry and layout as faithfully as possible, we used high-precision 3D data from the www.turboquid.com website as in our previous study^1^. The train considered, and its triangulated surface geometry in STL format, are presented in Fig. 12. The dimensions of the passenger coach are as follows: length L = 15.5 m; depth W = 2.5 m; and height h = 2.6 m.

**Figure 13:**
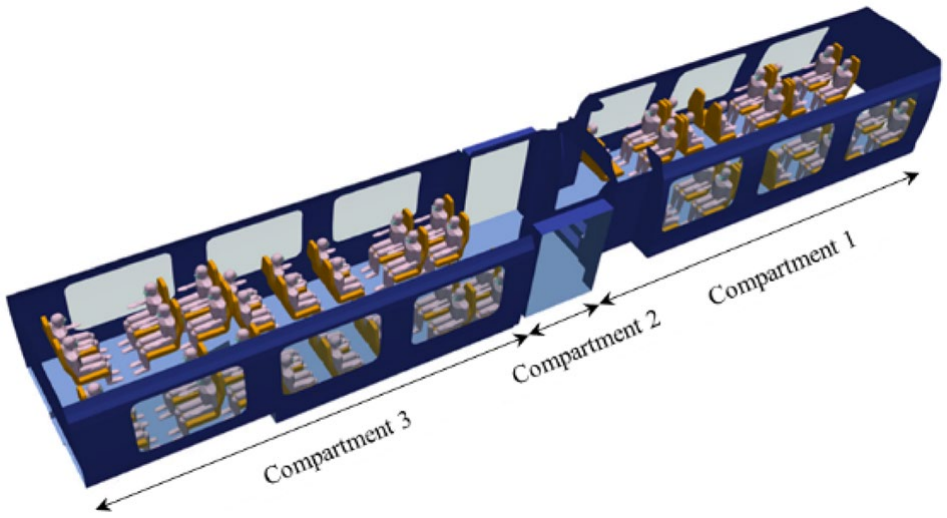
Geometry of the coach occupied by manikins and identification of the compartments.

**Figure 14:**
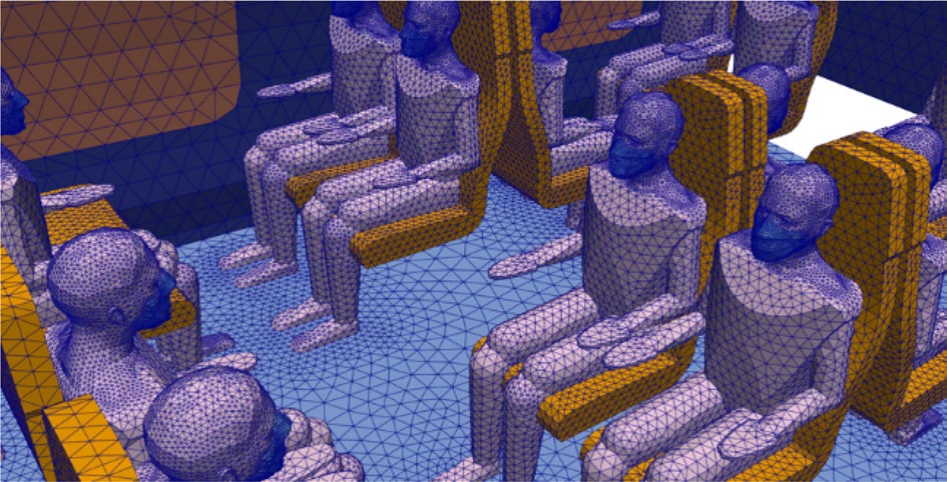
Representation of the surface mesh of the coach walls and of some masked manikins.

In addition, we have integrated into the 3D digital twin of the coach a number of human manikins whose bust and (masked or non-masked) head were presented earlier, but this time their full body is present. The final geometry of the CFD mock-up of the coach is shown in Fig. 13. It consists of three compartments whose seat occupancy rates by passengers are 92% for compartment #1, 0% for compartment #2 (coach entry and exit) and 100% for compartment #3. The CFD mesh has about 10 million tetrahedral cells; this shape was chosen because it is well suited to complex geometries. The minimum cell size is between 0.5 cm (on the masks of the manikins) and 3 cm (on the rest of the manikins and on the seats). The maximum size of the cells is 5 cm to 10 cm (on the walls of the coach). Regarding the mesh of the railway coach populated with unmasked or masked manikins, at least 99.9% of the cells have a skewness of less than 0.85 and most of them have a minimal skewness of less than 0.001, which is a proof of the quality of the mesh. Figure 14 zooms in on the triangular surface mesh on the walls of the coach and on some masked passengers.

In our previous article^1^, we considered passengers without masks among whom an infected passenger was breathing and coughing, thereby emitting particles of different sizes. Here, we account for the influence of mask use for the same dissemination events involving the pathogenic biological agent. The turbulent flow in the coach is generated by the ventilation taking place from bottom to top and from the ends of the coach to slots located in the ceiling of the coach. This ventilation mode was chosen because it is frequently encountered. It could, however, be amended without difficulty to account for any other types of ventilation. The steady flow due to the ventilation is established first, before the dissemination events begin. These preliminary aerodynamic results are, of course, not affected by whether or not the passengers wear a mask; these results were presented in our previous article^1^ and are therefore not reproduced here. In the next step, the simulated flow takes into account the local influence of breathing or coughing passengers. These unsteady computations are coupled with those of the dispersion of expectorated particles. The space and time distribution of the particles depends on their size, the flow in the coach (which is modified locally by the dissemination events, particularly the cough) and by the more or less well-fitted wearing of the mask. More details on flow and dispersion simulations are given in the “Methods” section of this article.

### Simulation results for breathing in the railway coach

Hereafter, we present the results of the simulations in the railway coach in the presence of breathing manikins, one of which is assumed to exhale droplets carrying the SARS-CoV-2 virions or other pathogens.

### Dispersion with the use of a perfectly fitting mask

Here we focus on the dispersion of the 1-µm particles emitted by the infected manikin with a perfectly fitted mask. It is worth noting that the position of the infected manikin in the coach is identical to that defined in the previous CFD study^1^ with non-masked manikins. In this numerical study as in the previous one, the duration of the simulated sequence is 600 s, which is sufficient for the droplets to spread throughout compartment #1 of the railway coach and to approach the stationary regime between production of droplets by the breathing of the infected manikin (in particular when it is masked), distribution of the droplets in the compartment and evacuation of droplets by the ventilation of this same compartment, as illustrated in the figures to follow.

**Figure 15:**
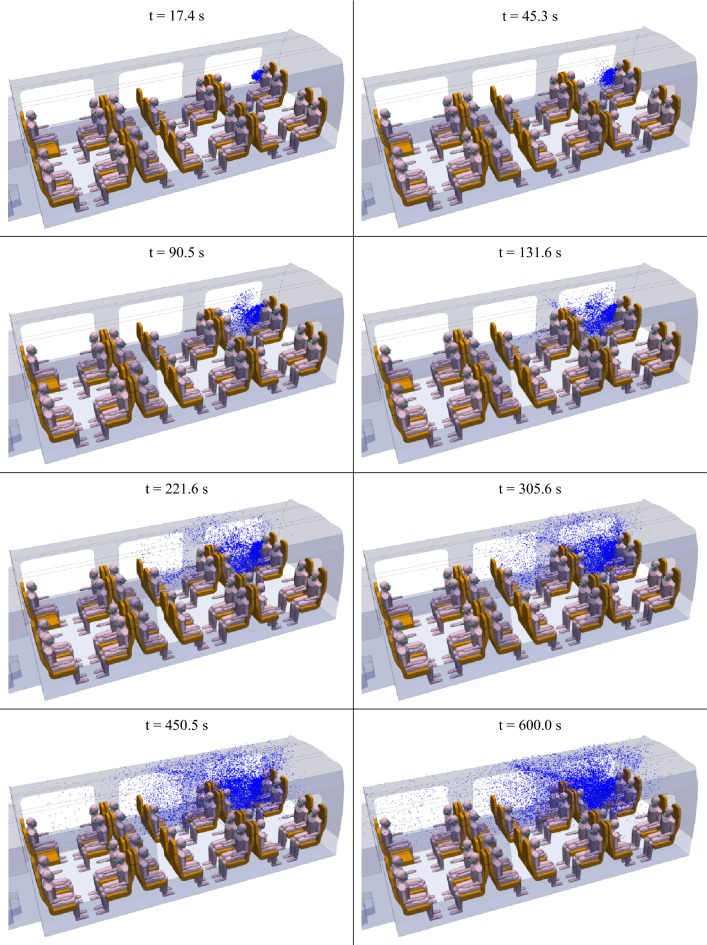
Distribution of droplets with a diameter of 1 µm, emitted by the infected manikin during its breathing at different times (t = 17.4 s; 45.3 s; 90.5 s; 131.6 s; 221.6 s; 305.6 s; 405.5 s; and 600 s) (left to right, and bottom to top).

Figure 15 shows the evolution of the spatial distribution of the droplets in the coach at different times between t = 17 s and t = 600 s. The propagation of droplets under the influence of the airflow generated by ventilation is thus observed over a relatively long period of time. Initially, for approximately 1 min, the droplets remain localised in the immediate vicinity of the source manikin (the disseminator), because the initial momentum of the breath of air and the droplets exhaled through the mask is particularly weak. This effect of mask use was already highlighted in the study of the manikin head and bust alone. The mask greatly reduces the velocity at which droplets are expelled, whether during a cough or, as here, during breathing. In the following minute, under the effect of the airflow, the droplets end up reaching the manikins opposite to the source manikin (t = 90.5 s to t = 131.6 s). After a few minutes (from t = 221.6 s), the droplets are also transmitted by the airflow to the row of four seats adjacent to that in which the source manikin is located. While a few droplets diffuse into the rest of the coach up to the central zone of compartment #2, we note that, in the simulated sequence, most of the droplets remain in the area of the two groups of four seats near the source manikin (that of the source manikin and the one immediately downstream), the other droplets being taken up by the air extraction system. As when no mask is worn^1^, the spread of the droplets is confined to the side where the disseminator is located. This is due to the ventilation configuration that separates the rows to the right and left.

The number of droplets exhaled by the infected manikin, i.e. injected into the simulation sequence of 600 s, is 29,048, and the number of droplets re-aspirated through the manikin mask during the inhalation phase (for the same simulation sequence) is 1068. The latter represents about 2% of the total number of droplets.

**Figure 16:**
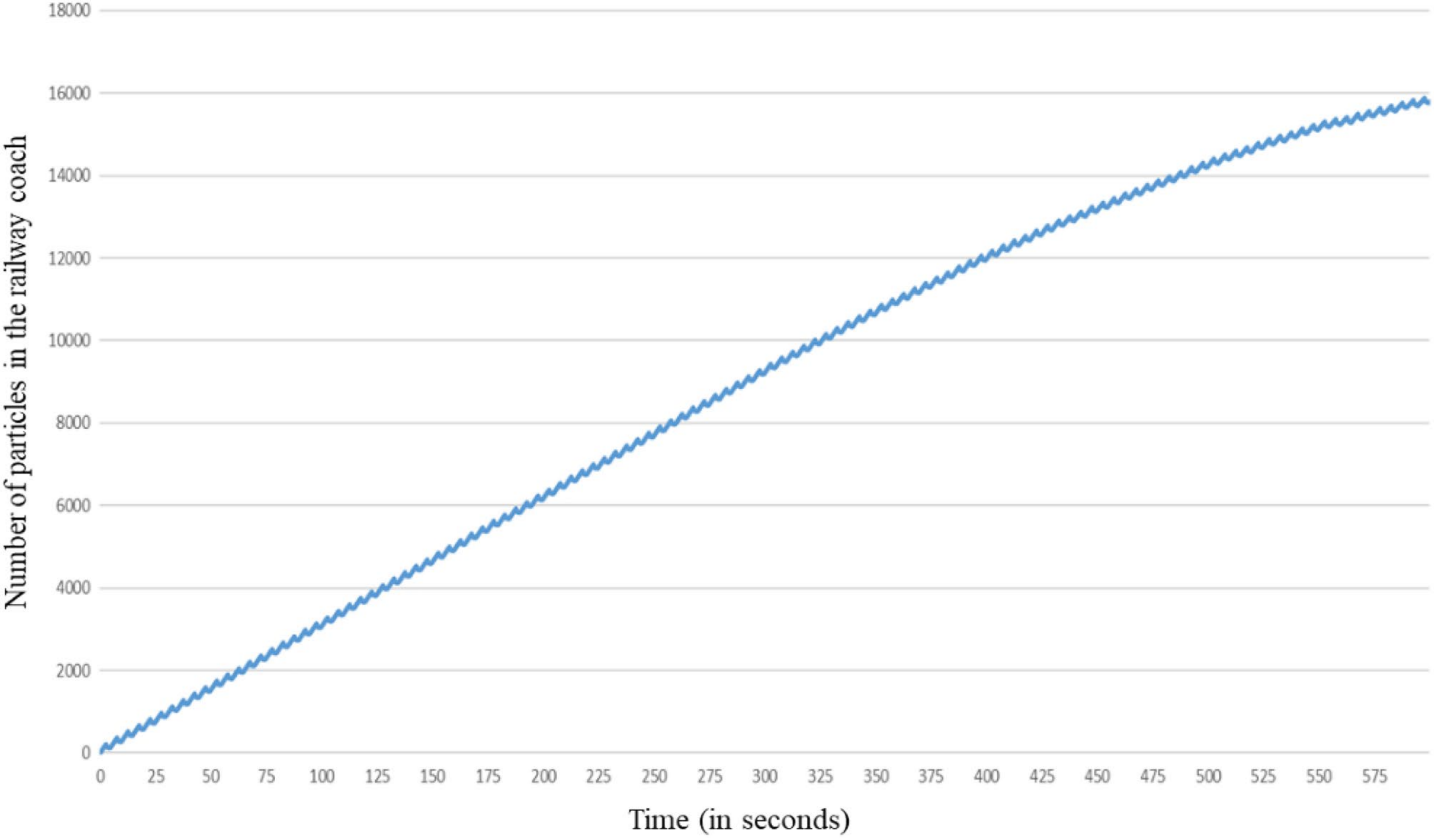
Evolution over time of the number of droplets remaining in the railway coach when the disseminator wears a mask.

Figure 16 shows the evolution of the number of droplets present in the coach during the simulated sequence of 600 s. After an initial linear increase due to the injection of droplets by the disseminator at a constant rate, the slope of the curve diminishes from about 400 to 500 s. Then, the evolution is again linear with a lower slope. The break in slope is due to the aspiration by the air extraction system located on the ceiling of the coach of a fraction of the droplets suspended in the air.

### Comparison of dispersion with and without mask use

Here we compare the dispersion of aerosols with a diameter of 1 µm produced by the breathing of the infected manikin depending on whether it is masked or not. The position of the source manikin is identical in both cases. The results in the case of non-masked passengers are taken from our previous work^1^.

**Figure 17:**
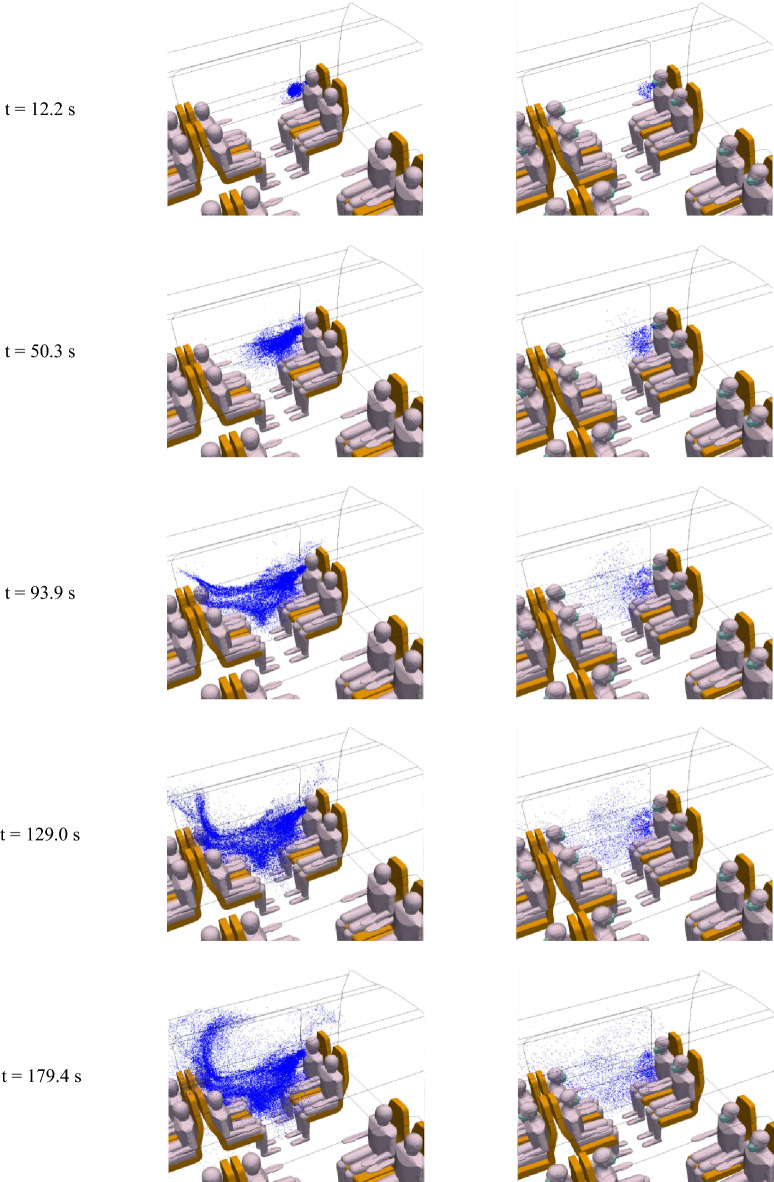
Distribution of 1-µm droplets emitted by the infected manikin during its breathing without mask use (left column) and with mask use (right column) at different times (t = 12.2 s; 50.3 s; 93.9 s; 129.0 s; and 179.4 s) (left to right, and bottom to top).

Figure 17 shows the comparison (on the left without mask use, on the right with mask use) of the distribution of droplets in the coach at different times between t = 12.2 s and t = 179.4 s. We immediately note the much greater number of droplets in suspension when the disseminator does not wear a mask. In particular, these results illustrate the role played by the mask in retaining a large proportion of the droplets. We also observe the slightest initial momentum of the droplets close to the face of the masked manikin. This once again demonstrates the important role that mask use plays in reducing the velocity of droplet expulsion. Droplet projection is much more pronounced, and the potential contamination of the passengers facing the disseminator occurs more quickly, when the disseminator does not wear a mask.

**Figure 18:**
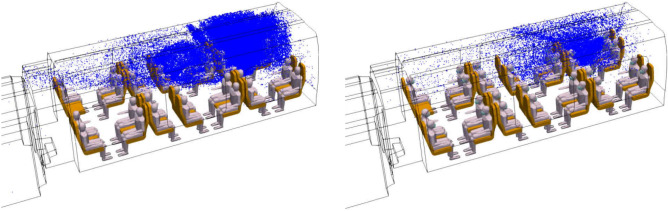
Distribution of 1-µm droplets emitted by the infected manikin without mask use (left) and with mask use (right) at the end of the simulated breathing sequence (t = 594 s).

Figure 18 also very clearly shows that at the end of the simulated sequence, the spatial extension of the droplets in the railway coach is significantly less if the disseminator manikin is masked compared to the situation where it does not wear a mask.

**Figure 19:**
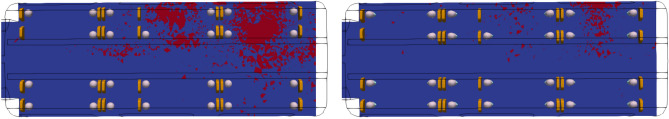
Cells (in red) containing at least one droplet, in a sectional plane at manikin face height, at the end of the simulated sequence with a disseminator wearing no mask (left) and wearing a mask (right).

One output of the Lagrangian dispersion simulation is the volume fraction of particles in each cell of the mesh. By multiplying this quantity by the volume of each cell, then dividing the result by the volume of a particle with a diameter of 1 µm, we can visualise the number of droplets in each cell of the mesh. Figure 19, which focuses on a sectional plane at the level of the faces of the manikins, shows the cells (in red) that contain at least one droplet at the end of the simulated breathing sequence. This visualisation makes it possible to observe the lesser extension of the droplets when the infected manikin is masked.

**Figure 20:**
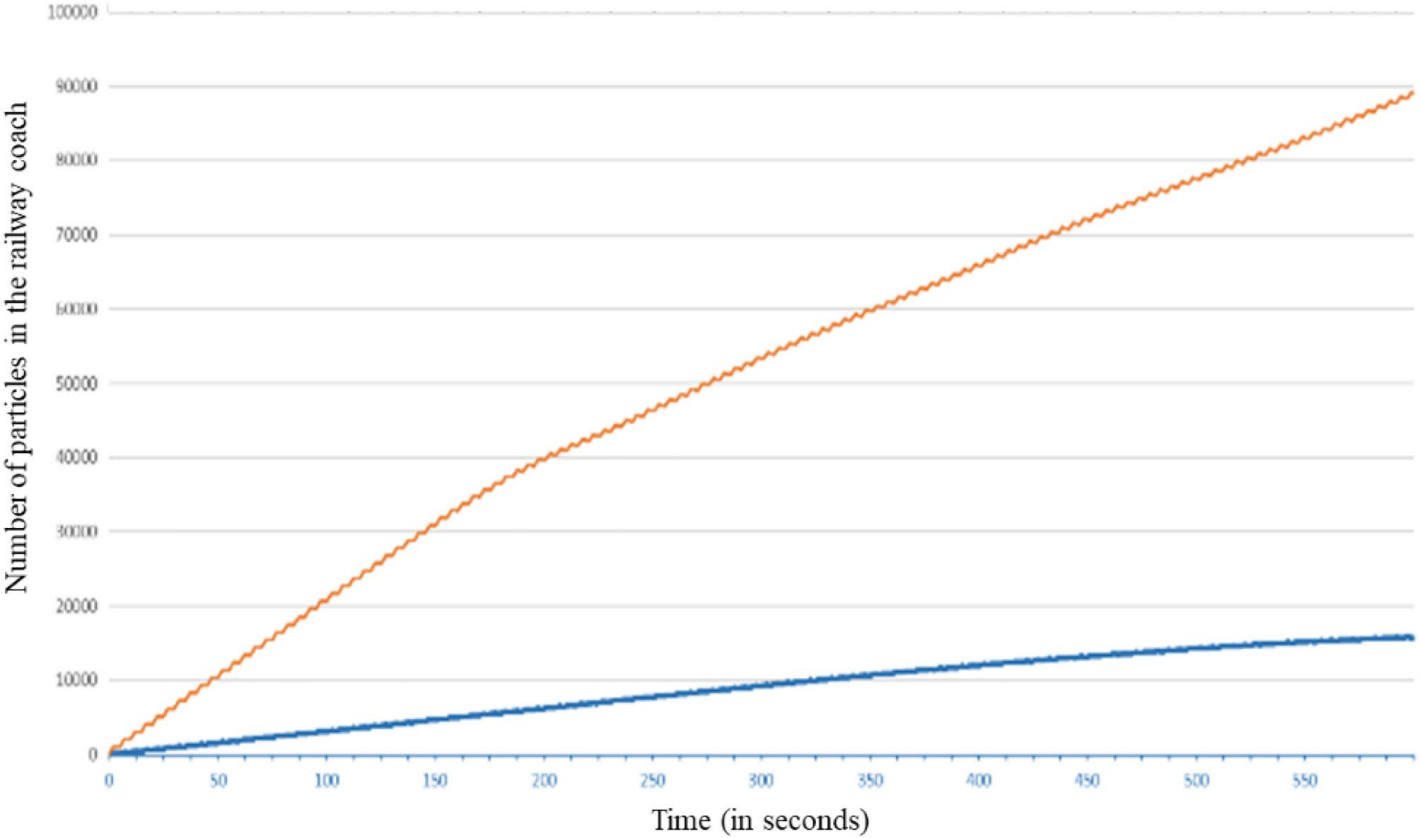
Evolution over time of the number of particles in the railway coach when the disseminator wears a mask (in blue) or does not wear one (in orange).

Figure 20 shows the evolution of the number of droplets present in the railway coach during the simulated sequence of 600 s, with and without mask use by the disseminator. It can be clearly observed that wearing a mask drastically reduces the number of droplets in suspension in the coach, thereby statistically reducing the risk of contamination. We also note the break in slope, which reflects the aspiration of a fraction of the droplets by the air extraction system; this break intervenes earlier when the disseminator is non-masked (t = 200 s approximately) than when the disseminator is masked (t = 300 s approximately). This effect comes from the greater initial momentum of the breath of air and the droplets when no mask is worn, which has the effect of bringing the particles more quickly and in greater quantity near the ceiling and the air extractions there (see for example Fig. 17 at t = 179.4 s for another illustration of this phenomenon). We found that the ratio of the number of particles present in the coach when the disseminator wears no mask, as compared to when a mask is worn, increases over time. At the end of the simulated breathing sequence, this ratio is around five, i.e. the inner volume of the railway coach contains six times more droplets carrying virions if the infected passenger does not wear a mask. Of course, this result depends on the semi-confined space considered, its ventilation, the position of the infected passenger who produces droplets and the filtration efficiency of the mask (see the “Methods” section for the assumptions made on this subject). It is also interesting to note from Fig. 20 that, in the case where the disseminator wears a mask, a steady state is reached at the end of the 600 s simulation, i.e. the number of droplets present in the coach becomes constant. This is not the case when the disseminator does not wear a mask, and it can be observed in Fig. 20 that the number of droplets in the coach continues to increase.

All of the previous results correspond to the wearing of a perfectly adjusted mask by the infected passenger who disseminates the pathogenic agent. For the sake of brevity, the case of the leaky mask is not presented in this article. This is an intermediate case between wearing a mask perfectly and not wearing a mask at all. In the case of a leaky mask, the flow and dispersion phenomenology is close to that of a well-fitting mask. In fact, a significant reduction in the velocity of the air and of the particles emitted through the mask is observed. The velocities through the leaks at the level of the nose, the cheeks and the chin are also low, and the effect of the breath of air directed towards the passenger in front of the disseminator is greatly reduced. The droplets tend to remain near the head of the disseminator, and although they end up being transported by the flow generated by the ventilation of the coach, they mostly remain in the area of the four passengers including the disseminator and in the row of four seats adjacent to that of the disseminator. With the properly worn mask as with the leaky mask, the particles have less of a tendency to occupy the entire interior volume of the railway coach. It therefore appears that wearing a mask is desirable in all cases, including when the mask does not fit perfectly on the face.

## Discussion

The research work presented in this article was begun in 2020 in the context of the Covid-19 pandemic, with the aim of illustrating the possibilities provided by CFD to both better understand and better control the airborne transmission of infectious agents such as SARS-CoV-2. In practice, we were interested in the flow of air inside the semi-confined, ventilated space of a railway coach as well as in the dispersion of droplets and drops produced by passengers who are breathing and coughing. Thus, we created the 3D digital twin of a railway coach occupied by human manikins and carried out simulations with the well-known, validated CFD model known as Code_SATURNE, which is presented in the “Methods” section. An initial study^1^ considering non-masked, disseminating passengers produced promising results and was extended by developing a parametric model to take into account the wearing of surgical masks by either infected or uninfected passengers. This model relates to the dynamics of breathing and coughing and the filtration efficiency of the mask upon exhalation and inhalation, by considering the diameter of the expectorated particles and by distinguishing between the case in which the mask is perfectly fitted and that in which the mask is worn loosely with leaks between the face of the wearer and the mask itself. The assumptions and parameters of the model were determined through an extensive bibliography dealing with particles produced by humans and also with the use of surgical masks. The 3D numerical study was carried out in two stages. First, dispersion simulations were performed by considering the head and bust of a manikin immersed in a neutral atmosphere in order to test the fitted mask and leaky mask models and check the consistency of our numerical results with regard to the experimental results available in the literature. Next, manikins wearing a mask more or less correctly, especially the manikin assumed to be infected by the pathogenic agent, were introduced into the digital twin of the railway coach, and flow and dispersion computations were performed. The number and distribution of the droplets produced by the disseminator manikin in the inner volume of the coach were compared depending on whether the disseminator manikin was wearing a mask or not.

The main achievements of our numerical study are summarised hereafter:

- We have carried out an up-to-date review of scientific data on the modes of transmission of pathogenic biological agents, the production of particles by humans (especially through breathing and coughing, and also, while not presented in the article, speaking, singing or sneezing), and the influence of masks, in particular surgical masks, on the spread of the expectorated particles. The data have been used to infer a model of the filtration efficiency of a surgical mask and the airflow around it. It is worth noting that the hypotheses formulated with regard to breathing with or without a mask and with regard to the filtration efficiency of the mask upon the exhalation or inhalation of drops and droplets may be easily adapted in the future should new scientific publications suggest an update is required. Data collected for dissemination events other than coughing such as sneezing will be used in future simulations.
- We have illustrated the capability and value of CFD, notably through the use of Code_SATURNE software, for simulating the 3D turbulent flow and dispersion of particles carrying infectious agents in ventilated inner spaces with complex geometries. Dispersion computations performed using either an Eulerian model or a Lagrangian model led to similar results not compared in this article for the sake of brevity. Associated with the validation of Code_SATURNE, this consistency between results gives us confidence in our use of the CFD tool. We have also demonstrated the fundamentally different aerodynamic behaviour of droplets, which constitute an aerosol that follows the flow almost perfectly, and that of drops, which move away from the flow by the effect of inertia and the predominance of sedimentation through their deposition. The 3D numerical results were post-processed to obtain visually meaningful and didactic graphical representations such as the temporal evolution of the spatial distribution of the drops and droplets. This aspect is important when it comes to exporting scientific results, for example to health authorities that do not have knowledge of modelling and simulation, as was one of the purposes of this study.
- Dissemination simulations involving a human manikin wearing a surgical mask more or less well fitted to its face clearly illustrate the benefit of the mask in reducing both the number of airborne droplets in the railway coach and the extent of the area where droplets are present. This is all the more true if the mask is worn correctly, but it remains applicable even if the mask is worn in more realistic conditions, i.e. loosely and with leaks around the face. This reduction originates from the filtration of the exhaled air by the mask and the decrease in the velocity of the breath of air moving through the mask. In this respect, it is interesting to point out that, in the case of cough, the effect of the mask, whether more or less well worn, is to stop the drops and slow down the droplets, which reduces the spectrum of particles associated with this dissemination event and brings it closer to the situation of respiration where only droplets are emitted at low speed. In our simulations, the influence of the mask is not only demonstrated, but quantified, which to the best of our knowledge has never been attempted before. While this quantification depends on the ventilated space under consideration, the location of the disseminator and the filtration efficiency of the mask, we have at our disposal a CFD-based method to carry it out. We must recall the well-known fact that masks, even when correctly worn, allow droplets to pass through them, which means that they only limit and do not prevent infection by the virions of the Covid-19 disease or any other respiratory disease. Another fact that may be less known is that the filtration efficiency of droplets by surgical masks upon inhalation is low and that such masks therefore do not constitute individual protection devices. Through this application to public transport, we have shown the potential of numerical simulation for understanding the phenomenology and, subsequently, for determining the means of limiting the dissemination of airborne infectious agents. It is not just this specific example but the general feasibility of this type of work and the value of the overall concept that we wanted to demonstrate.

From a scientific perspective, several follow-ups are envisaged to complete the work carried out.

- This study implements the k-epsilon turbulence model in the RANS approach of Code_SATURNE, a validated software used to adequately depict the average transport and dispersion of particles carried by the turbulent flow. In further development, large-eddy simulations (LES) could be considered as an alternative to the RANS approach at the cost of significantly longer calculations. While we expect to obtain similar averaged results, LES would provide more detailed information about the space and time fluctuations of the turbulent flow and of the phenomena affecting the particles such as propagation, mixing and dilution. The respective advantages of the RANS and LES methods could be established by comparing the results obtained by each method.
- Some phenomena have not been considered in our numerical study; these include mass transfers (evaporation of liquid particles) and processes specific to aerosols (nucleation, agglomeration, etc.). Modelling these phenomena would be useful to study the role of ambient temperature and ambient humidity in influencing the transmission of the virions and the contamination of human beings. It can already be noted that when droplets evaporate, their diameter decreases until a dry residue is obtained, which corresponds to one or a few virions. The size of a virion is of the order of 100 nm. The dynamics of particles between 0.1 and a few micrometers is very comparable. Therefore, taking into account the evaporation of droplets should not fundamentally modify their spatial and temporal distribution.
- Our article focuses on the dissemination of infectious agents emitted during respiratory events by masked or unmasked individuals only from a fluid mechanics perspective. Afterwards, it would obviously be very interesting to integrate within our model biological characteristics of the SARS-CoV-2 virions or other airborne infectious agents, subject to acquiring the relevant information held by specialists in this field. This would constitute an excellent opportunity for collaboration between fluid mechanics experts and biologists. For example, one could assess the viral load contained in the liquid particles, determine the number of droplets and, therefore, virions inhaled by people in the vicinity of a spreader (in our case, the passengers in the railway coach) or account for the evolution of the pathogenicity of the virions, once the particles have dried up, whether they are in suspension or deposited on surfaces.
- The manikins present in the digital twin of the railway coach could be rendered more diverse (presence of men, women and children). The disseminator could be made to sneeze or emit droplets when speaking, a process that has not yet been looked at. In our study, we preferred to consider a plausible number of particles produced by different dissemination events and to treat different particle sizes separately. Indeed, to date, a great variety of observations have been made, without consensus, regarding the spectrum of drops and droplets generated by coughing or sneezing (for example). That said, there would be no difficulty in specifying (for modelling purposes) the spectrum of particles produced during the different types of dissemination events (breathing, coughing, etc.) to make the simulations even closer to real life.
- Moreover, if we consider the diversity of data published in the literature both on the spectrum of droplets and drops produced in respiratory events and on the characteristics of masks (surgical or otherwise), it would be interesting to conduct a sensitivity study on these parameters. This would be done at the cost of many calculations but would make it possible to determine which parameters are the most influential and which are the most unfavourable cases in terms of aerial dissemination of infectious agents. Moreover, these multiple calculations would constitute an approach to take uncertainties into account. For example, the results could thus be expressed in the form of the probability of finding droplets or drops at a given time and place.

Also, on a practical level, this work could initiate several very useful and instructive developments.

- The railway coach considered in our simulations is representative in terms of geometry and ventilation. Still, there exist many models of trains and coaches, and the simulations could be repeated by considering data associated with different “real” coaches. It should also be emphasised that the ventilation conditions in the computations carried out are realistic, but probably do not strictly correspond to the conditions actually encountered in public transport. In particular, we assumed that the air injected by the ventilation system is devoid of the infectious agent. However, it seems that, in most cases, the air injected into public transport coaches is, mainly for reasons of energy economy, partially composed of recycled air. If it does not include a sterilising treatment or an effective filtering system, for example a high-efficiency particulate absorbing (HEPA) filter, air recycling has an effect of accelerating the dispersion of aerosols in the coach. This could be accounted for in future simulations.
- The effect of modifications to the internal configuration of this coach, or any other coach, on the spatial and temporal distribution of the particles could be considered. Examples of modifications are to turn the seats by positioning them facing the windows or to install separating partitions between the rows of seats. It would thus be possible to determine whether a particular configuration can limit the dissemination of the particles (and to what extent), and whether it would be worthwhile to implement.
- The method developed in our work could easily be transposed to other types of masks (in particular, the FFP2 mask made compulsory in some countries at the time of the Covid-19 pandemic) by adapting the mask model and its parameters. Modelling could be also extended to other means of transport such as cruise ships or airplanes. For the latter it is worth noting that there are experimental data such as those of TRANSCOM / AMC Commercial Aircraft Cabin Aerosol Dispersion Tests, which could provide further validation of Code_SATURNE. Moreover, modelling could be applied to other types of more or less confined, ventilated spaces intended for collective use, such as performance halls, restaurants, nurseries, school rooms or company “open space” offices, to name a few.
- Just as generally, numerical simulation should make it possible to deduce instructive results about how the ventilation design and the internal configuration of premises could be optimised to lessen the transmission of airborne pathogens. It should also enable the assessment of the criticality of gathering people, some of whom are carriers of infectious agents, in closed or semi-closed spaces and the development of recommendations applicable to SARS-Cov-2 virions or other respiratory pathogens.

## Methods

Our study is based on physical modelling and numerical simulation, which are used here to replace experimental tests. Indeed, these would be very difficult to set up in a real railway coach with real passengers wearing a mask or not (although, of course, full-scale experiments will always be necessary). For this, we used a CFD model that has been extensively validated, in particular in the case of turbulent flows that are generated by a ventilation system and that transport particles of different diameters. The study involved a series of stages comprising choices related to the modelling, the search for relevant available data, the development of a digital twin of a railway coach occupied by human manikins wearing masks, the implementation of flow and dispersion simulations and, finally, the post-processing of the results to make them easily exploitable. The physical modelling considered the turbulent flow, the dispersion of particles produced by manikins breathing or coughing and, more specifically, the influence of the mask on respiratory events in terms of both the flow and the filtration of particles. The numerical options mainly dealt with the type and refinement of the meshing and with parameters such as the time step. Otherwise, a number of data were necessary to run the simulations, including the geometry and ventilation of the rail coach, the occupancy of the coach by the passengers, and the characteristics of the particles generated by the passengers in the course of respiratory events. Some of the modelling and data aspects are commented on in this section, which first provides essential information on the simulations carried out in this study, then focuses successively on the ventilation in the railway coach and on the particle source terms associated with the breathing and coughing of human beings.

### CFD simulations with code_SATURNE

All of the simulations presented in this article were carried out using the Code_SATURNE^45^ (version 7.0) general-purpose, open-source CFD computational tool developed by the R&D division of the French electricity supplier EDF and the Atmospheric Environment Teaching and Research Centre (CEREA) in France. Code_SATURNE is a finite volume code using a fully co-located arrangement for all variables on structured or unstructured 2D or 3D meshes with e.g. tetrahedral or hexahedral cells. It has several numerical solvers, based on prediction and correction steps, for laminar or turbulent, steady or unsteady, uncompressible or compressible, isothermal or non-isothermal, non-reactive or reactive, monophasic or multiphasic flows.

Code_SATURNE provides several approaches to the flow dynamics, principally the Reynolds-averaged Navier–Stokes (RANS) technique and large-eddy simulations (LES). In this study, we chose to use the former approach with the “k-epsilon” turbulence model^46^. This closure model solves the equations of the turbulent kinetic energy (k) and of its dissipation rate (epsilon). It is known to be robust and well adapted to moderate Reynolds number ventilation flows. Moreover, it gives results in quite short spans of time. It is thus widely used for scientific and industrial applications^47,48^. More specifically, we implemented an update of the standard k-epsilon model, namely the k-epsilon linear production model, which assumes that the turbulent production source term is linear with respect to the flow strain. This modification of the k-epsilon model has been demonstrated to improve predictions of turbulent flows in numerous cases^49^. In association with the turbulence model, a “scalable” or “hybrid” wall function was used to account for the buffer region and the laminar sub-layer when approaching walls. It is a blended wall treatment that is well adapted to moderate Reynolds number flows and k-epsilon simulations^46^.

Code_SATURNE has the advantage of proposing both Eulerian and Lagrangian transport and dispersion models. In the former, particles are treated as a phase carried by the airflow, and their volumetric concentration is obtained by solving a transport and dispersion equation (if the particles have different diameters, they can be sorted by classes with as many equations solved as classes of particles). In the latter, particles are considered individually accounting for their own diameters, and their trajectories are determined by solving as many equations of motion as there are particles. Both approaches are coupled to the turbulent flow simulations through the forces acting on the particles and by the average and fluctuating components of the velocity. In a previous study^1^, we demonstrated that the results provided by Code_SATURNE using either approach were similar. Thus, in this study, we carried out simulations only with the Lagrangian model.

The equations solved by Code_SATURNE are not shown in this article as they were presented exhaustively in our previous study^1^**,** to which the interested reader is referred for more information.

Code_SATURNE has been developed under quality assurance procedures and is used both in industry and for academic research. Efforts were invested in qualification processes in fields as diverse as aerodynamics (including pollutant dispersion), nuclear thermal-hydraulics, engines, combustion or process engineering. Code_SATURNE has been thoroughly validated against measurements in a very large number of academic case studies exhibiting analytical solutions and industrial applications implying complex geometries^50–53^. Some of the numerous validation cases relate to internal flows and configurations close to the numerical study presented in this article, such as the ventilation of premises comprising several rooms possibly undergoing fire, the distribution of chemical species in a gas turbine, and the trajectories and deposition of pulverised coal particles in a furnace. These case studies correspond to turbulent regimes and imply the resolution of the Navier–Stokes equations in RANS formalism using the k-epsilon turbulence model. In the last two cases mentioned, the Eulerian transport equation and the Lagrangian dynamics equations of particles in size ranges from a few microns to a few tens of micrometers are solved respectively. More details about Code_SATURNE validation were given in the previous article^1^.

In this article, we present two types of simulations. On the one hand, we consider the head and bust of a human manikin immersed in quiescent air. Flow was generated uniquely by the particle dissemination events (breathing and coughing), which were modelled dynamically, meaning that the flow and dispersion simulations were performed simultaneously in unsteady state. On the other hand, we consider the situation of human manikins corresponding to passengers seated in a commuter train. The flow was generated by the mechanical ventilation of the rail coach and modified locally by the breathing or coughing of the passengers. In this case, a stationary simulation of the flow due to ventilation in the coach was first carried out. Then, unsteady simulations of flow and dispersion were performed, taking into account the ventilation of the rail coach as well as the respiration and the emission of particles by a supposedly infected manikin. One can note that in the simulations carried out, heat and mass transfers were not considered, an assumption that will be lifted in our next work (see the perspectives in the Discussion section).

Regarding the numerical method, we implemented the SIMPLEC velocity–pressure coupling algorithm for the flow simulations. The stationary flow generated by the mechanical ventilation in the rail coach was computed using a pseudo-steady CFL-limited solver with a space- and time-varying time step. The unsteady flow locally perturbed by a dissemination event (cough or respiration) was computed along with the dispersion of particles. The time step is a crucial parameter to ensure the robust convergence and the accuracy of the simulations. It has to be chosen carefully depending on the mesh spatial discretisation. The time step is constant and its value depends on the flow velocity. It is shorter for the faster events and longer for slower events. In the simulations around the head of the manikin, the time step is 0.01 s in case #1 without a mask or in case #3 with the leaky mask, and 0.1 s in case #2 with the perfectly fitting mask, as here the flow velocity is lower. In each case, the simulated sequence lasts for 30 s (coughing) or 120 s (breathing). In the unsteady simulations in the railway coach, the time step is 0.01 s, 0.05 s or 0.1 s depending on the configuration, and the simulated sequence lasts for 50 s (coughing) or 600 s (breathing).

Flow simulations in the rail coach were carried out using unstructured meshes of tetrahedral cells. Meshes with different geometrical characteristics and levels of refinement were benchmarked. The flow results were compared in order to evaluate the sensitivity of the numerical solution as a function of the mesh refinement. This analysis showed that the flow fields were almost identical from the finest mesh to the coarsest mesh, which was used to run the flow simulations and obtain the concentration results based on Lagrangian dispersion modelling. Results regarding the flow in the railway coach were presented and commented on in the previous study^1^. Therefore, they are not reported here.

The computer used for the simulations was equipped with a Bi-Xeon® Intel CPU processor with 2 × 40 hyper-threaded cores in the cases with only the manikin and with 2 × 16 hyper-threaded cores in the application to the coach populated with manikins. Tables 1 and 2 indicate the principal features and durations of the flow and dispersion simulations, respectively around the manikin and in the railway coach occupied by manikins.

**Table 1:** Main characteristics and durations of the simulations around the manikin.

	#1—No mask	#2—Fitted mask	#3—Leaky mask
Number of mesh cells	480,820	237,176	235,340
Simulations of the flow and particle dispersion generated by the cough			
Number of CPU cores	40	Not presented	40
Time step	0.01 s	Not presented	0.01 s
Sequence duration	30 s	Not presented	30 s
Computation duration	0.33 h	Not presented	0.16 h
Simulations of the flow and particle dispersion generated by breathing			
Number of CPU cores	20	40	40
Time step	0.01 s	0.1 s	0.1 s
Sequence duration	120 s	120 s	120 s
Computation duration	1.76 h	0.16 h	0.63 h

**Table 2:** Main characteristics and durations of the simulations in the rail coach.

	#1—No mask	#3—Leaky mask
Number of mesh cells	4 million	9.8 million
Simulation of the flow due to mechanical ventilation in the railway coach		
Number of CPU cores	10	40
Computation duration	12.4 h	27.2 h
Simulations of the flow and particle dispersion generated by the cough		
Number of CPU cores	15	Not presented
Time step	0.01 s	Not presented
Sequence duration	50 s	Not presented
Computation duration	71.6 h	Not presented
Simulations of the flow and particle dispersion generated by breathing		
Number of CPU cores	20	70
Time step	0.1 s	0.05 s
Sequence duration	600 s	600 s
Computation duration	71.4 h	28.2 h

### Modelling of the ventilation in the railway coach

We endeavoured to model the ventilation in the digital twin of the coach in such a way as to approach what can be found in a real carriage. While the characteristics of the ventilation considered in our model are common, they may be different in other rail coaches. Still, it would be quite easy to modify the ventilation system in order to account for alternative blowing and extracting air vents corresponding to different models of carriages. The ventilation features relate to the conditions of air supply and extraction, that is to say the location, shape and dimensions of the supply and extraction vents and the flow rates through these vents. The data chosen in our simulations were inspired by a review of air conditioning and ventilation in trains, and by the geometry files of the selected rail coach.

**Figure 21:**
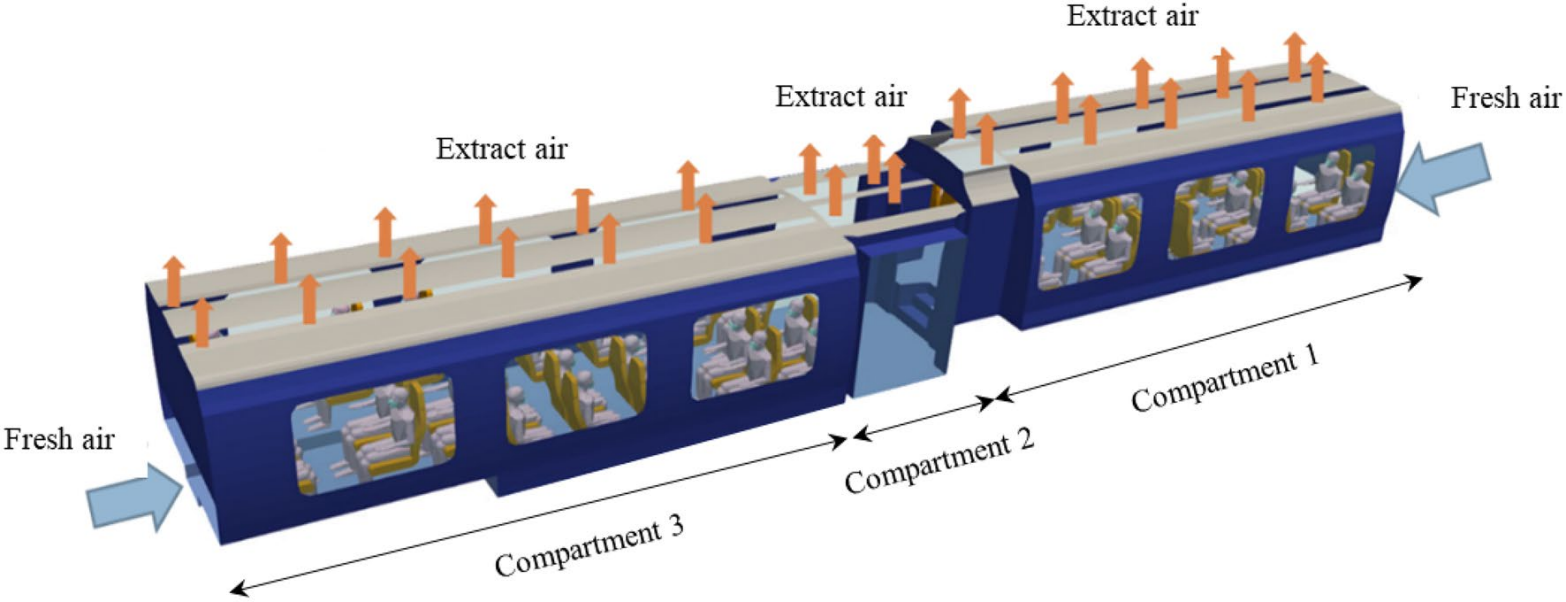
Layout of the ventilation in the digital twin of the rail coach with incoming and outgoing airflows.

The ventilation system is organised into zones corresponding to the compartments represented in Fig. 21, which sketches the layout of the ventilation in the rail coach. We assume that only fresh air is supplied from the outside to each end of the coach at imposed velocities and flow rates. Air extraction is performed through slits in the roof of each compartment. In compartments #1 and #3 occupied by passengers, the exit velocities and flow rates are imposed, while in compartment #2, air is extracted at atmospheric pressure, resulting in a balance of overall airflows between the compartments. In this way, the ventilation involves the air in the entire space of the coach. The airflow is directed globally from the bottom to the top of the coach. We adjusted the air extraction rates of compartments #1 and #3 to have air moving from the ends to the middle of the coach, with 50% of the air being extracted through compartment #2. The velocities of the supplied and extracted air are less than 0.2 m.s^−1^, which corresponds to the “gentle” ventilation prescribed by railway operators to ensure comfortable conditions for passengers. The renewal rate of the air in each compartment is computed using the flow rate supplied to the compartment. It is equal to 8.7 or, in other words, the air is refreshed every 7 min, which is better than the minimum recommended for ventilated public spaces. The average residence time is obtained by dividing the volume of each compartment by the air flow rate supplied to the compartment. Ventilation data used as inlet or outlet boundary conditions of the aerodynamic simulations are gathered in Table 3.

**Table 3:** Ventilation data for the digital twin of the rail coach.

Compartment #1	27.34 m^2^	Compartment #3	30.49 m^2^
Air supply unit		Air supply unit	
Supply surface (one vent)	1.098 m^2^	Supply surface (one vent)	1.338 m^2^
Supply velocity	0.060 m.s^−1^	Supply velocity	0.055 m.s^−1^
Supply flow rate	0.066 m^3^.s^−1^	Supply flow rate	0.074 m^3^.s^−1^
Air extraction unit		Air extraction unit	
Extraction surface (slots)	1.427 m^2^	Extraction surface (slots)	1.550 m^2^
Extraction velocity	0.023 m.s^−1^	Extraction velocity	0.024 m.s^−1^
Extraction flow rate	0.033 m^3^.s^−1^	Extraction flow rate	0.037 m^3^.s^−1^
Air extraction deficit	0.033 m^3^.s^−1^	Air extraction deficit	0.037 m^3^.s^−1^
Geometrical residence time	833 s	Geometrical residence time	855 s
Air renewal rate	8.7	Air renewal rate	8.7

### Modelling of the dissemination events producing drops and droplets

**Figure 22:**
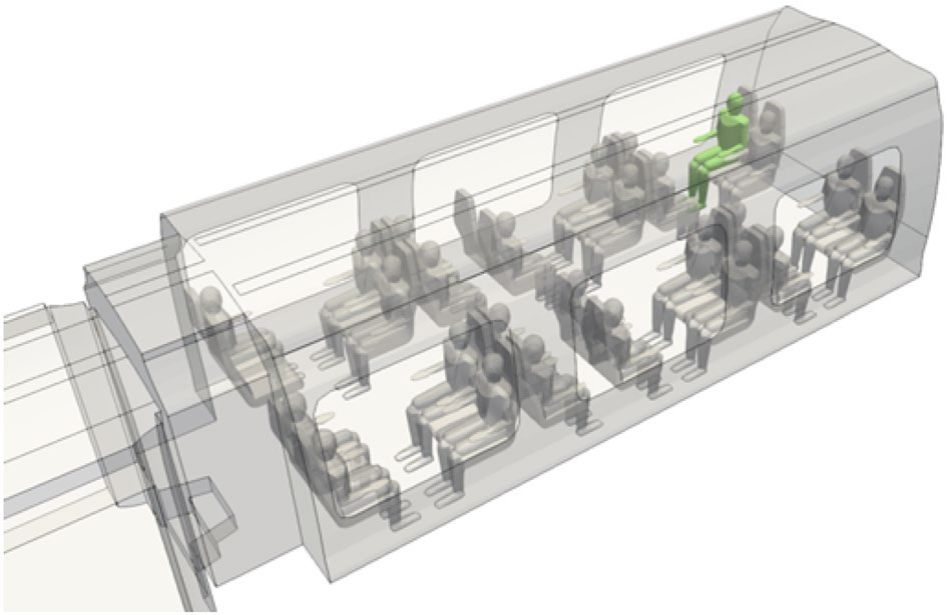
Location of the manikin wearing a mask (or not) and disseminating the infectious agent by breathing.

Typically, coughing and breathing are respiratory events causing the dissemination of droplets and drops likely to carry pathogenic biological agents such as the virions of the Covid-19 disease. Though they are both expectorations, coughing and breathing are different in many aspects. A cough leads to a single release of short duration. Of course, more than one cough, as occurs with a coughing attack, could be considered, with several coughs simulated one after the other. In contrast, exhalation leads to a periodic release related to the respiration cycle. Without a mask, the initial momentum of the expectoration is much higher for the cough than for the exhalation. Wearing a mask reduces the velocity of particle-carrying air passing through the mask, and the air velocity is also low through any leaks around the face. Another difference between coughing and exhalation without a mask is the size of the particles produced. While coughing may lead to a full spectrum of droplets and drops, exhalation produces micrometric droplets. The particle size spectrum produced by coughing can vary considerably from person to person, and depending on the type and intensity of the cough. For this reason, we decided to consider particles separately over a wide range of sizes, from 1 to 1000 µm in aerodynamic diameter. Of course, wearing a mask has a significant effect on the spectrum and number of particles that pass through the mask, which we will see in the following. Interestingly, the mask makes coughing and breathing more closely resemble each other in terms of both aerodynamics and the sizes of particles likely to be emitted into the air. We must also mention that the dissemination events reported here were considered independently in our simulations. In the digital twin of the railway coach, the disseminator manikin wearing a mask is assumed to be seated in compartment #1 of the coach and occupy the position indicated in Fig. 22, just as in the previous study^1^ with non-masked manikins. This location is arbitrary and could easily be modified.

**Figure 23:**
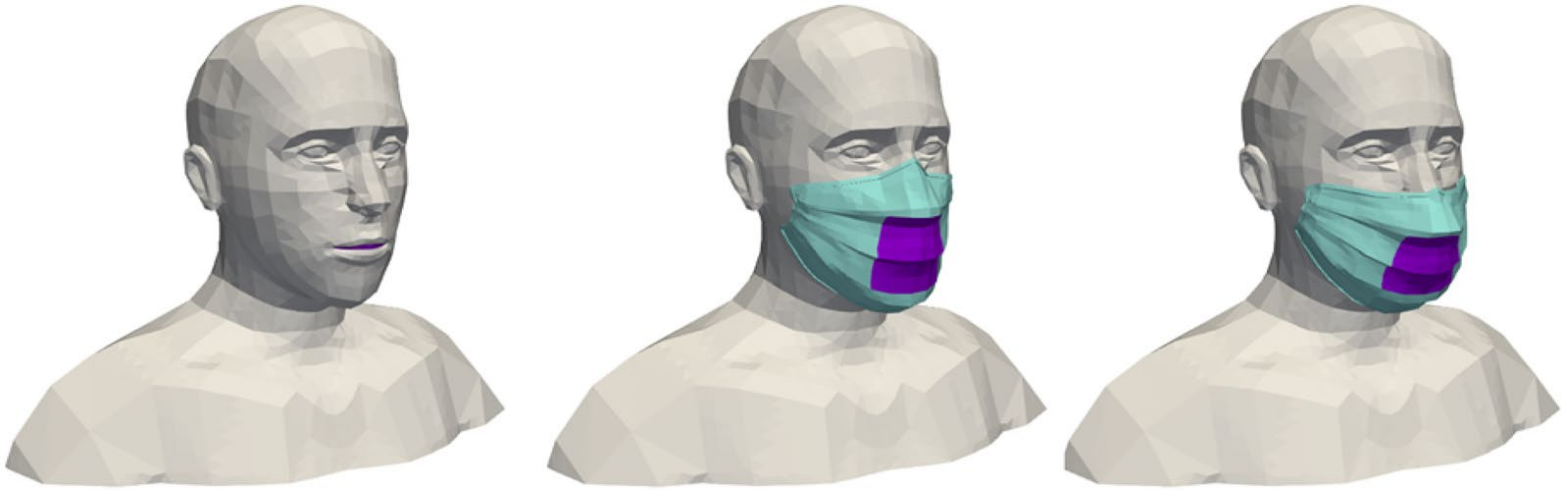
Representation of the air inlet through the mouth (without mask, left), and also through the mask (in purple) with a mask perfectly fitted on the face (centre) or with a leaky mask (right). Images created by the authors with Paraview 5.8.1 (www.paraview.org).

First, in case #1, we considered a cough, as well as breathing through the mouth, without a mask. The air inlet surface of the domain is the mouth of the manikin. Then, in cases #2 and #3, we dealt with the use of more or less well-fitting masks. The air inlet surface of the domain consists of the part of the mask represented in purple in Fig. 23 and the leaks (if any). Regarding the number of droplets passing through a surgical mask, the literature gives sometimes contradictory results on filtration by surgical masks and very few results on leaks between the face and the mask. For this reason, we adopted a pragmatic, realistic approach in order to estimate the quantities dispersed through the mask and at the level of the leaks based on a choice made from among the scientific publications that we identified. The reader may note that the quantities defined are adjustable and that the values adopted are to be considered as parameters that can easily be varied in the simulations.

### Modelling of respiration and associated production of particles with or without a mask

**Figure 24:**
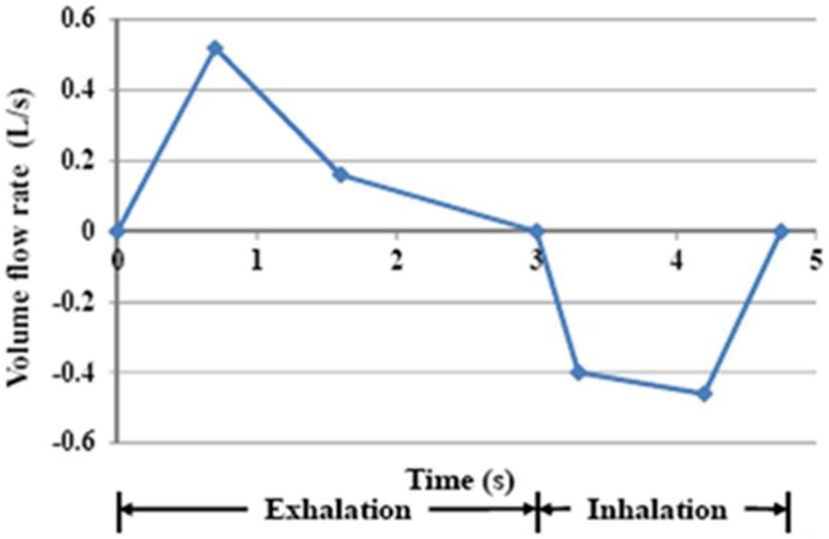
Evolution of the basic air flow rate (in L.s^−1^) during the human respiratory cycle.

**Figure 25:**
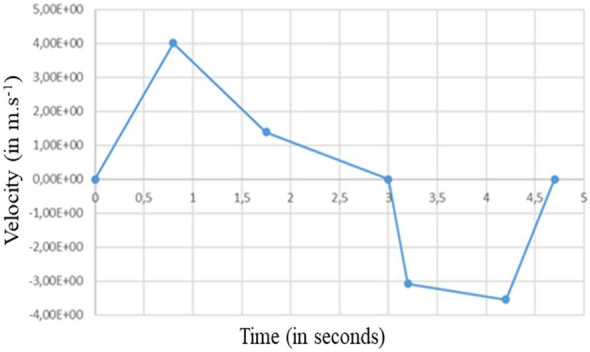
Evolution of the air velocity (in m.s^−1^) through the mouth of the manikin in case #1.

**Figure 26:**
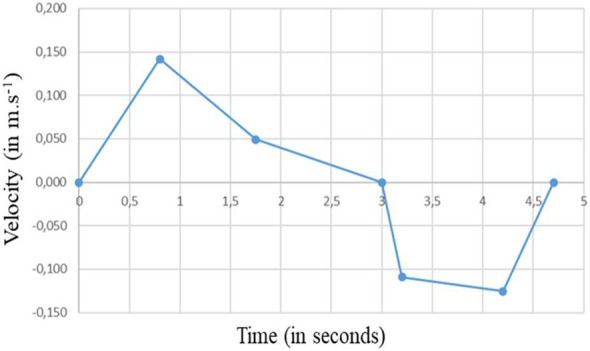
Evolution of the air velocity (in m.s^−1^) through the mask worn by the manikin in case #2.

**Figure 27:**
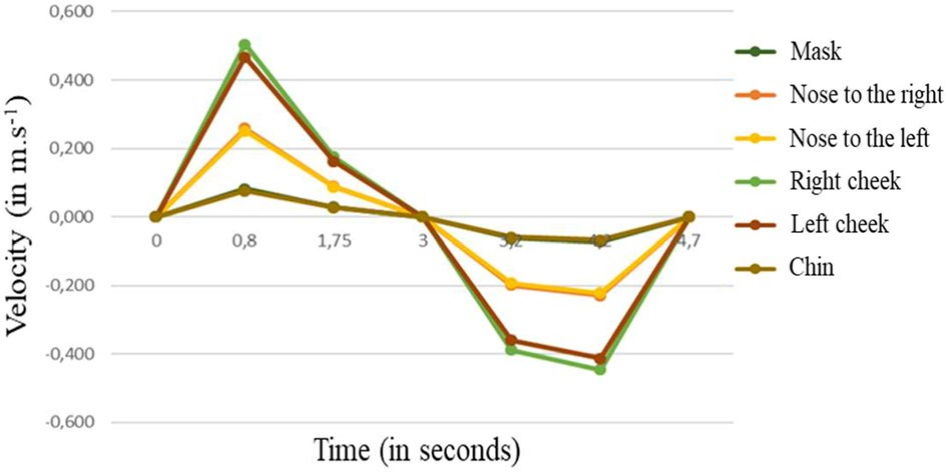
Evolution of the air velocity (in m.s^−1^) through the mask and leaks around the face of the manikin in case #3.

We used the results of the literature^35,36^ to model the respiratory flow rate of a human being. The air cycle is defined linearly by pieces and comprises successive phases of exhalation and inhalation as presented in Fig. 24. In case #1, knowing the surface of the mouth of the manikin, we calculated the velocity of expiration and inspiration through the mouth, as shown in Fig. 25. The breath of air is directed orthogonally to the mouth of the manikin with an angle of 15° beneath the horizontal direction. In cases #2 and #3, we adapted the air velocities through the mask and the leaks (if any) according to the inlet surfaces of the mask and the leaks. We used the literature results^35,36^ to determine the distribution of leaks around the edge of the mask when it is not optimally adjusted. The air jets through the mask and the leaks (if any) are oriented orthogonally to the facets of the surface mesh of the mask and the leaks. Using mass conservation with the same total flow rate to distribute through the various inlet surfaces, Table 4 indicates the air inlet surfaces and flow distribution in case #1 (mouth only), case #2 (mask only) and case #3 (mask and leaks). Figures 26 and 27 show the evolution of the air velocity through the mask and leaks (if any), for cases #2 and #3, respectively.

**Table 4:** Air inlet surfaces and flow distribution in cases #1, #2 and #3.

	Surface (in m^2^)	Share of the flow rate
Case #1		
Mouth	1.2980 × 10^–4^	100%
Case #2		
Mask	3.6619 × 10^–3^	100%
Case #3		
Mask	3.2039 × 10^–3^	50%
Nose to the right	2.5198 × 10^–4^	12.5%
Nose to the left	2.5861 × 10^–4^	12.5%
Right cheek	1.0302 × 10^–4^	10%
Left cheek	1.1138 × 10^–4^	10%
Chin	3.4013 × 10^–4^	5%

As for breathing, we simulated the dispersion of droplets with a diameter of 1 µm, because it is accepted that droplets of less than 10 µm in diameter exhibit the same overall behaviour in the air. Moreover, exhalation is generally not accompanied by the production of drops, and at any rate, drops of more than 10 µm in diameter are correctly filtered by surgical masks. Given the diameter of the droplets, their deposition on surfaces was considered to be nil or negligible and was therefore not taken into account. In case #1 (no mask), the number of particles injected into the domain was equal to 1000 in the exhalation phase. In the following inhalation phase, droplets may possibly be re-inhaled through the mouth of the manikin if they are close enough, and depending on local velocity conditions. In case #2 (perfectly fitted mask), we considered the efficiency of droplet filtration through the mask upon exhalation to be equal to 75% based on results from the literature^43^. With this filtration efficiency, the number of droplets exhaled through the mask was equal to 250. As in case #1, droplets can be re-aspirated by the manikin through the mask during the inhalation phase. In this phase, we assumed on the basis of our review that all the particles that were likely to be inhaled passed through the mask, which reflects an inhalation filtration efficiency of 0%. In case #3, we accounted for the same efficiency of droplet filtration through the mask upon exhalation as in case #2, i.e. 75%, and 250 droplets exhaled per cycle. Furthermore, the literature results^28^ led us to consider an overall exhalation filtration efficiency of 56%, taking into account leaks on the perimeter of the mask. With this data and the distribution of leaks in Table 4, we defined the number of droplets that pass through the leaks without being filtered, as shown in Table 5. As in the other cases, droplets can be re-aspirated by the manikin through the mask during the inhalation phase. Filtration efficiency upon inhalation is taken to be equal to 0%.

**Table 5:** Distribution of the droplets exhaled through the mask and the leaks during a respiratory cycle in case #3.

Overall mask efficiency with leaks	56%
Number of particles passing through the mask	250
Number of particles passing through the leaks	191
Nose to the right	48
Nose to the left	48
Right cheek	38
Left cheek	38
Chin	19

### Modelling of a cough and associated production of particles with or without a mask

For the simulation of one cough, we considered the airflow conditions through the mouth of the manikin when it does not wear a mask and at the level of the mask and leaks (if any) when it wears a mask. In case #1 (with no mask), the air inlet velocity into the domain from the mouth is 4.5 m.s^−1^ for 0.5 s. The breath of air is directed orthogonally to the mouth of the manikin with an angle of 15° beneath the horizontal direction. In case #3 (with the leaky mask), we calculated the air velocity during a cough lasting 0.5 s based on the data previously used for breathing (surfaces and distribution of the flow rates as in Table 4) in order to respect mass conservation. The air velocities through the different inlets of the simulation domain are indicated in Table 6.

**Table 6:** Air velocity through the mask and the different leaks upon dissemination by coughing in case #3.

Zone	Velocity (in m.s^–1^)
Mask	0.08
Leaks along the nose	0.25
Leaks along the cheeks	0.50
Leaks along the chin	0.07

Regarding the particles emitted when coughing without a mask (case #1), we considered the four diameter classes of 1 µm, 10 µm, 100 µm and 1000 µm, with 10,000 particles per class as in our previous study^1^. With a leaky mask (case #3), the situation is very different. As can be observed in Fig. 10, the drops of 100 µm in diameter settle almost immediately, and the drops of 1000 µm in diameter follow ballistic trajectories. Thus, we assume that these drops are projected into the mask where they are filtered, thereby remaining blocked. We considered the droplets of 1 µm and 10 µm, and added droplets of 50 µm, with 10,000 droplets per class. The 50 µm droplets exhibit an intermediate behaviour between that of finer and larger particles. Like the drops, they are filtered by the mask, but they tend to go through the leaks around the face of the manikin. Finally, only droplets of 1 µm and 10 µm are able to cross the mask. The number of droplets exhaled through the mask and through the leakage zones is presented in Table 7. It was established assuming an overall filtration efficiency of 56%^28^ for the mask and the same distribution among the leaks^35,36^ as the one used for breathing given in Table 4. Case #2 (the perfectly fitting mask), not presented in this article, involves only droplets with diameters of 1 and 10 µm. The velocity of the droplets through the mask is 0.16 m.s^−1^. It is obtained from the air velocity of 4.5 m.s^−1^ when coughing, multiplied by the ratio of the mouth surface to the mask surface, which is 0.0354 (see Table 4). Thus, case #2 resembles case #3, but without leaks.

**Table 7:** Distribution of particles exhaled during coughing through the mask and the leaks in case #3.

Zone	Number of particles		
	1 µm	10 µm	50 µm
Mask	2500	2500	0
Leaks along the nose	480 (on each side)	480 (on each side)	480 (on each side)
Leaks along the cheeks	380 (on each side)	380 (on each side)	380 (on each side)
Leaks along the chin	190	190	190

## Data Availability

All data from the study can be requested by contacting Patrick Armand.
